# TREM-1 activation is a potential key regulator in driving severe pathogenesis of enterovirus A71 infection

**DOI:** 10.1038/s41598-020-60761-5

**Published:** 2020-03-02

**Authors:** Siti Naqiah Amrun, Jeslin J. L. Tan, Natasha Y. Rickett, Jonathan A. Cox, Bernett Lee, Michael J. Griffiths, Tom Solomon, David Perera, Mong How Ooi, Julian A. Hiscox, Lisa F. P. Ng

**Affiliations:** 1 Singapore Immunology Network, Agency for Science, Technology and Research, Singapore, Singapore; 2 National Institute of Health Research, Health Protection Research Unit in Emerging and Zoonotic Infections, University of Liverpool, Liverpool, United Kingdom; 3 Institute of Infection and Global Health, University of Liverpool, Liverpool, United Kingdom; 4 Walton Centre NHS Foundation Trust, Liverpool Health Partners, Liverpool, United Kingdom; 5 Institute of Health and Community Medicine, University Malaysia Sarawak, Sarawak, Malaysia; 6 Department of Paediatrics, Sibu Hospital, Sibu, Sarawak, Malaysia; 7 Department of Biochemistry, Yong Loo Lin School of Medicine, National University of Singapore, Singapore, Singapore

**Keywords:** Viral infection, Transcriptomics, Viral pathogenesis

## Abstract

Hand, foot and mouth disease (HFMD), caused by enterovirus A71 (EV-A71), presents mild to severe disease, and sometimes fatal neurological and respiratory manifestations. However, reasons for the severe pathogenesis remain undefined. To investigate this, infection and viral kinetics of EV-A71 isolates from clinical disease (mild, moderate and severe) from Sarawak, Malaysia, were characterised in human rhabdomyosarcoma (RD), neuroblastoma (SH-SY5Y) and peripheral blood mononuclear cells (PBMCs). High resolution transcriptomics was used to decipher EV-A71-host interactions in PBMCs. Ingenuity analyses revealed similar pathways triggered by all EV-A71 isolates, although the extent of activation varied. Importantly, several pathways were found to be specific to the severe isolate, including triggering receptor expressed on myeloid cells 1 (TREM-1) signalling. Depletion of TREM-1 in EV-A71-infected PBMCs with peptide LP17 resulted in decreased levels of pro-inflammatory genes for the moderate and severe isolates. Mechanistically, this is the first report describing the transcriptome profiles during EV-A71 infections in primary human cells, and the potential involvement of TREM-1 in the severe disease pathogenesis, thus providing new insights for future treatment targets.

## Introduction

Hand, foot and mouth disease (HFMD) is a febrile illness that predominantly affects infants and young children, and is characterised by rash and blisters on the hands, mouth, feet and bottoms^1–3^. Outbreaks of HFMD are caused by human enterovirus group A members (HE-A), mainly coxsackieviruses A16, A6 and A10, and enterovirus A71 (EV-A71)^1,4,5^. While often benign and self-limiting^2^, the disease can cause cardiopulmonary and neurological complications such as myocarditis, brainstem encephalitis, aseptic meningitis, and neurogenic pulmonary oedema, which can be fatal^6,7^. The severe manifestations of HFMD are often associated with cases of EV-A71 infections, rather than coxsackievirus A16^8^.

EV-A71 belongs to the *Enterovirus A* species, *Enterovirus* genus, from the *Picornaviridae* family^9,10^. The virus has a positive-sense RNA genome of 7.4 kb and encodes for four structural (VP1–4) and seven non-structural (2A-C and 3A-D) proteins^9^. First isolated in California, USA, in 1969^11^, the virus is transmitted via the oral-foecal route, saliva and respiratory secretions^12^. Based on the sequence analysis of VP1 gene, the virus can be broadly categorised into six genogroups: A, B, C, D, E and F^7,10,13^. Whereas genogroup A contains the Californian prototype virus BrCr, genogroups B and C consist of various strains, and are further categorised into sub-genogroups B0-B5 and C1-C5^10^. B and C genogroups are globally distributed, whereas D, and E and F are limited to India and Africa respectively^7,10^. After the almost total eradication of poliovirus, EV-A71 is now noted as one of the most prevalent neurotropic enteroviruses, and is especially endemic in the Asia-Pacific region^14–17^. Currently, the reasons for the emergence of neurological diseases from EV-A71 infections are still unknown^1^, and this poses a significant public health problem as there are no antivirals available^18^. Two inactivated EV-A71 vaccines have been approved for use by China’s Food and Drug Administration (FDA), but they have not gained widespread adoption into immunisation programs globally^19,20^.

In this study, clinical EV-A71 isolates associated with differing levels of severity (mild, moderate, and severe) collected from an outbreak in 2006 in Sarawak, Malaysia^21^, were used to characterise and compare the infection kinetics, virus tropism and immuno-pathogenesis using *in vitro* and *ex vivo* models. In particular, infection kinetics and high-density transcriptomic profiling by RNA-sequencing (RNA-seq) of EV-A71-infected peripheral blood mononuclear cells (PBMCs) revealed several virus severity-dependent differences. Collectively, this is the first report describing the immune mechanisms involved during EV-A71 infections in primary human cells, and the identification of novel pathways, such as triggering receptor expressed on myeloid cells 1 (TREM-1) signalling, that are unique and specific to the severe EV-A71 isolate, and likely contributing to the severe manifestations of the disease.

## Results

### Clinical EV-A71 isolates are phylogenetically classified based on disease outcomes

**Figure 1:**
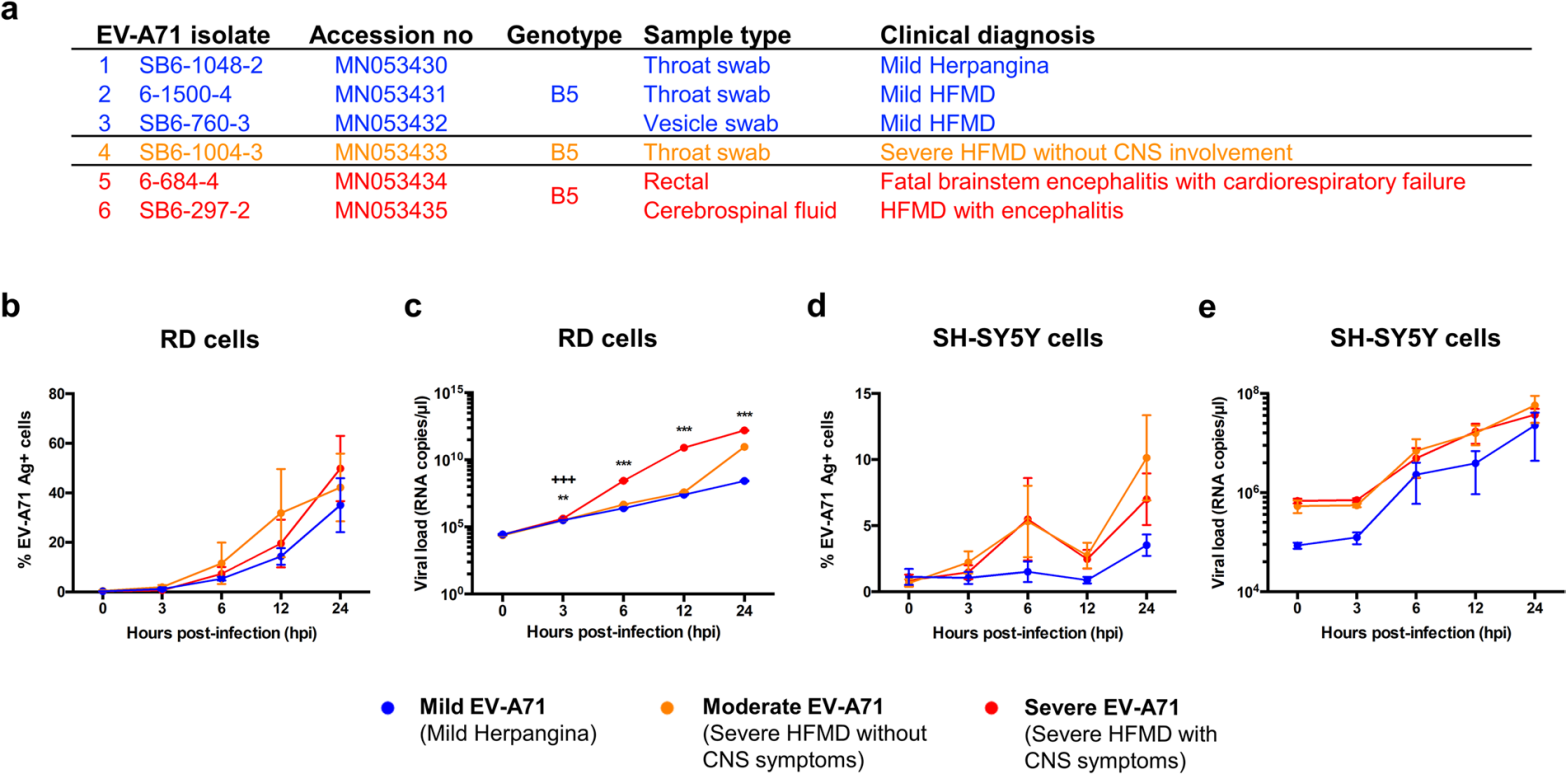
EV-A71 viral isolates from 2006 outbreak in Sarawak, Malaysia, and its viral kinetics in human rhabdomyosarcoma (RD) and neuroblastoma (SH-SY5Y) cells. (**a**) Clinical information about the viruses isolated including genotype, symptoms and area virus was isolated from. (**b**–**e**) RD and SH-SY5Y cells were infected with mild, moderate and severe isolates of EV-A71 at MOI 10 and 1 respectively, and harvested at 0, 3, 6, 12 and 24 hours post-infection (hpi). (**b,c**) Infection kinetics in RD cells via (**b**) quantification of VP1 antigen by flow cytometry and (**c**) viral load quantification of VP1 RNA by qRT-PCR. (**d**,**e**) Infection kinetics in SH-SY5Y cells via (**d**) quantification of VP1 antigen by flow cytometry and (**e**) viral load quantification of VP1 RNA by qRT-PCR. Data are presented as mean ± SEM and representative of three independent experiments. Statistical analysis was carried out with Kruskal-Wallis with Dunn’s multiple comparisons test to compare among EV-A71 isolates at the respective time-points (**p<0.01; ***p<0.001) [Mild vs Severe EV-A71 (*); Moderate vs Severe EV-A71 (+)].

Six EV-A71 isolates were identified from patients during an outbreak of HFMD in Sarawak, Malaysia, in 2006^22^. These patients had differing levels of disease severity, ranging from mild herpangina/HFMD, severe HFMD in the absence or presence of neurological complications, and one fatality (Fig. 1a, Supplementary Fig. S1a). However, alignment analysis of the different coding sequences of the virus revealed the isolates to be at least 92% identical to one another (Supplementary Table S1). Moreover, phylogenetic classification of the viruses based on the sequence of the VP1 gene identified them as belonging to the B5 sub-genogroup, and were closely related to strains circulating in Sarawak, Brunei, and Singapore from 2003 to 2008 (Supplementary Fig. S1b). Despite sharing 96% VP1 genetic similarity (Supplementary Table S1), all of the isolates, except isolate 3, were observed to be clustered according to disease severity in the phylogenetic tree (Supplementary Fig. S1b).

### Severe EV-A71 isolate infects human muscle RD cells to high levels

To investigate if the different EV-A71 clinical isolates have different infection profiles that could contribute to disease outcome, infection kinetics were compared in human muscle rhabdomyosarcoma (RD) cells and neuroblastoma SH-SY5Y cells at multiplicity of infections (MOI) 10 and 1, respectively. These cell lines have been extensively used in elucidating viral kinetics^7,23^ and pathogenesis of enteroviruses^24,25^. The infection kinetics across a time-course of 0, 3, 6, 12, and 24 hours post-infection (hpi) were determined by measuring infectivity parameters of percentage of intracellular VP1 antigen-positive cells by flow cytometry (Supplementary Fig. S2a) and levels of VP1 RNA load by qRT-PCR. In RD cells, the percentage of infected VP1+ cells among the EV-A71 isolates 1, 2 and 3 within the mild disease phenotype group were similar by 24 hpi (Supplementary Fig. S2b). Likewise, there were no significant differences between isolates 5 and 6 of the severe phenotype over the course of the time-points (Supplementary Fig. S2c). As such, isolates 1, 4 and 5 were chosen as representatives for further characterisation, and were defined as “mild”, “moderate”, and “severe” EV-A71 isolates respectively.

The severe isolate showed the highest level of infection in RD cells, followed by moderate and mild (Fig. 1b,c). The percentage of EV-A71 VP1 antigen in live cells assessed via flow cytometry showed that by 24 hpi, severe EV-A71-infected RD cells exhibited the highest levels of VP1+ cells, followed closely by moderate EV-A71, and mild EV-A71 (Fig. 1b). Severe EV-A71 also showed significantly higher virus replication compared to the mild isolate from 3 to 24 hpi, whereas moderate EV-A71 had higher VP1 RNA copies than mild EV-A71 only at 24 hpi (Fig. 1c). However, there were no significant differences in percentage of VP1+ cells and viral RNA copies among the three isolates at almost all time-points in SH-SY5Y cells (Fig. 1d,e).

### Primary human monocytes, B and T cells are the main targets of EV-A71

**Figure 2:**
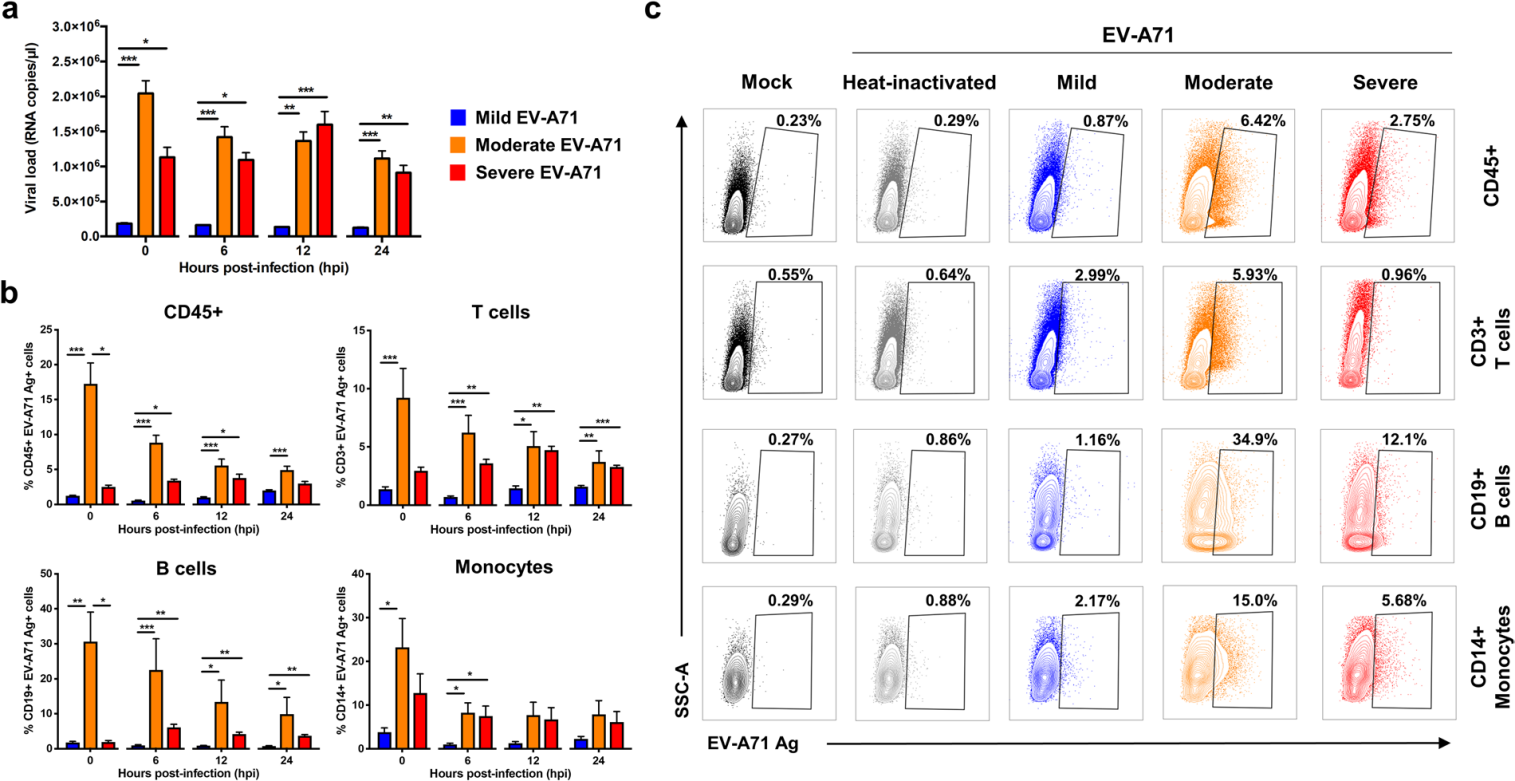
EV-A71 infection in primary human peripheral blood mononuclear cells (PBMCs). Human primary PBMCs (n = 5–9) were infected with mild, moderate, severe and heat-inactivated EV-A71 isolates at MOI 5, and harvested at 0, 6, 12 and 24 hpi for viral load quantification and flow cytometry. **(a)** Viral load levels were determined by qRT-PCR. **(b,c)** Percentage of EV-A71 Ag+ cells from total CD45+, CD3+ T cells, CD19+ B cells and CD14+ monocytes were determined via flow cytometry. **(b)** Bar graphs showing the levels of EV-A71 Ag+ cells from the different cell subsets. Data are presented as mean ± SEM. **(c)** Illustration of representative contour plots from one donor at 12 hpi. Statistical analysis was carried out with Kruskal-Wallis with Dunn’s multiple comparisons test to compare among EV-A71 isolates at the respective time-points (*p < 0.05; **p < 0.01; ***p < 0.001).

Infection kinetics of the different EV-A71 isolates were next explored in human PBMCs to assess if the associated disease severity affects the ability of the virus to infect the primary cells, which are more biologically relevant than cell lines. In this *ex vivo* model, healthy donor PBMCs were infected with mild, moderate, severe, and heat-inactivated forms of EV-A71 at MOI 5 over 0, 6, 12 and 24 hpi. PBMCs infected with moderate and severe isolates of EV-A71 had higher levels of viral VP1 RNA load compared to the mild isolate (Fig. 2a). The latter showed lower and limited viral replication throughout the course of infection, whereas moderate EV-A71-infected PBMCs had the highest viral load in the initial phase, before steadily decreasing over time (Fig. 2a). However, PBMCs infected with the severe isolate demonstrated increasing viral load levels till 12 hpi, before dropping off at 24 hpi (Fig. 2a). These observed trends were also consistent with the percentage of EV-A71 VP1 antigen detected from total CD45+ leucocytes (Fig. 2b,c).

To identify the specific subsets susceptible to EV-A71 infection, cells were further gated into the different populations: T cells, B cells, monocytes, and natural killer (NK) cells (Supplementary Fig. S2d). T and B lymphocytes, as well as monocytes, were the main susceptible subsets within the PBMCs (Fig. 2b,c). In contrast, NK cells showed limited and lower levels of infectivity (Supplementary Fig. S2e). Again, PBMCs infected with mild EV-A71 demonstrated an overall limited percentage of VP1 antigen within the three susceptible subsets (Fig. 2b), whereas moderate-infected PBMCs showed highest VP1 percentage in B cells, followed closely by monocytes, and T cells (Fig. 2b). On the other hand, PBMCs infected with the severe isolate displayed a slightly different trend, with the highest percentage of VP1 antigen observed in the monocytes, followed by B cells and T cells (Fig. 2b). Moreover, there was an increase in the percentage of VP1 antigen in the B and T lymphocytes populations at 6 hpi and 12 hpi respectively, whereas in the monocytes the percentage decreased at 6 hpi but was sustained throughout the course of infection (Fig. 2b).

### Transcriptomic profiling reveals a strong induction of host antiviral immune response upon EV-A71 infection

**Figure 3:**
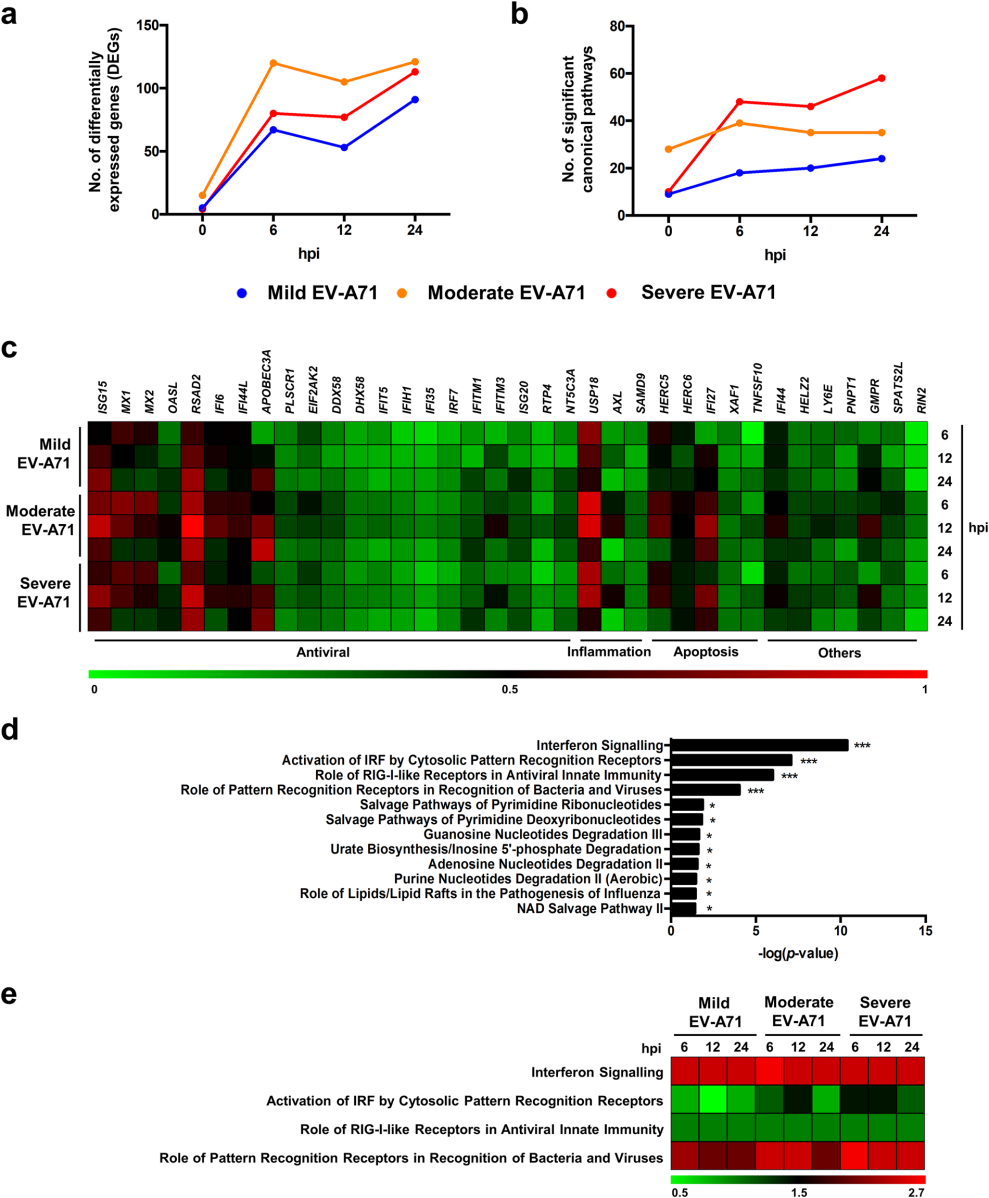
Transcriptomic profiling of human PBMCs during EV-A71 infection. Primary human PBMCs (n = 4) were infected with mild, moderate, severe and heat-inactivated EV-A71 isolates at MOI 5, and harvested at 0, 6, 12 and 24 hpi for transcriptomic analysis by RNA-seq. Results of infected conditions were compared to heat-inactivated control, and the log_2_-(fold change) values were analysed in Ingenuity Pathway Analysis (IPA). **(a,b)** Overall changes of significant **(a)** differentially expressed genes (DEGs) and **(b)** canonical pathways identified by IPA over time and in different isolates. **(c)** Heat-map showing normalised log_2_-(fold change) values of the common DEGs in all infected conditions of 6, 12 and 24 hpi. **(d)** Significant canonical pathways determined by IPA from the common DEGs in  **(c)** (*p < 0.05; ***p < 0.001). **(e)** Heat-map showing the activation *z*-score values of the top four canonical pathways as determined by IPA.

To identify the possible mediators responsible for the disease severity-dependent differences, RNA-seq was performed on samples of EV-A71-infected PBMCs from four donors to identify and quantitate mRNA abundance and changes throughout the course of infection at 0, 6, 12 and 24 hpi. Mapped genes of infected conditions were normalised to a heat-inactivated control virus at the respective time-points. The results showed that the number of significant differentially expressed transcripts (false discovery rate [FDR] < 0.05) were different across time-points as well as isolates (Fig. 3a). During the course of infection, the greatest change occurred from 0 to 6 hpi for all EV-A71 isolates, as indicated by the sharp increase in the number of differentially expressed genes (DEGs), and the amount of DEGs continued to show an overall increasing trend from 6 to 24 hpi (Fig. 3a). Moderate EV-A71 demonstrated the highest numbers of DEGs throughout infection, followed by severe EV-A71, and mild EV-A71 (Fig. 3a).

Significant DEGs of the respective isolates and time-points were analysed in Ingenuity Pathway Analysis (IPA) to identify functional pathways (Fig. 3b). The numbers of significantly enriched canonical pathways [-log(*p*-value) > 1.30] were highest for severe EV-A71-infected PBMCs, especially from 6 to 24 hpi (Fig. 3b), despite being presented with fewer differentially expressed transcripts than moderate-infected PBMCs (Fig. 3a). PBMCs infected with mild EV-A71 displayed the lowest number of functional pathways (Fig. 3b).

In order to characterise the cellular responses elicited upon EV-A71 infection, transcripts that were common among the three isolates at time-points 6 to 24 hpi were identified and further analysed. Regardless of isolate severity, a total of 36 genes were differentially expressed during EV-A71 infection (Supplementary Table S2), and these genes are known to be involved in the antiviral interferon response, inflammatory processes, as well as in apoptosis (Fig. 3c). Nonetheless, the proportion of fold change for most of the genes differed across time and among the EV-A71 isolates. Notably, PBMCs infected with moderate EV-A71 showed a stronger response than the mild and severe isolates, specifically interferon-stimulated gene 15 (*ISG15*), viperin (*RSAD2*), ubiquitin specific peptidase 18 (*USP18*), interferon alpha inducible protein 27 (*IFI27*), and guanosine monophosphate reductase (*GMPR*) (Fig. 3c). Next, in order to identify the common functional pathways, the 36 genes were further analysed in IPA. The results yielded 12 significant canonical pathways (Supplementary Table S3) and expectedly, the antiviral response and virus recognition formed the top four pathways (Fig. 3d,e). Based on the activation *z*-scores generated by IPA, the interferon and RIG-I like signalling pathways were equally activated among the EV-A71 isolates, and across time (Fig. 3e). The pathway involving virus recognition by pattern recognition receptors (PRR) was most significantly activated in severe EV-A71-infected conditions at 6 hpi that persisted till 24 hpi (Fig. 3e). However, a different pattern was observed for mild and moderate EV-A71 infections, in which the activation of this pathway became less pronounced over time (Fig. 3e). Similar observations were also seen for the interferon-regulatory factor (IRF) activation pathway (Fig. 3e).

### Activation of TREM-1 pathway is specific to severe EV-A71 infection

**Figure 4:**
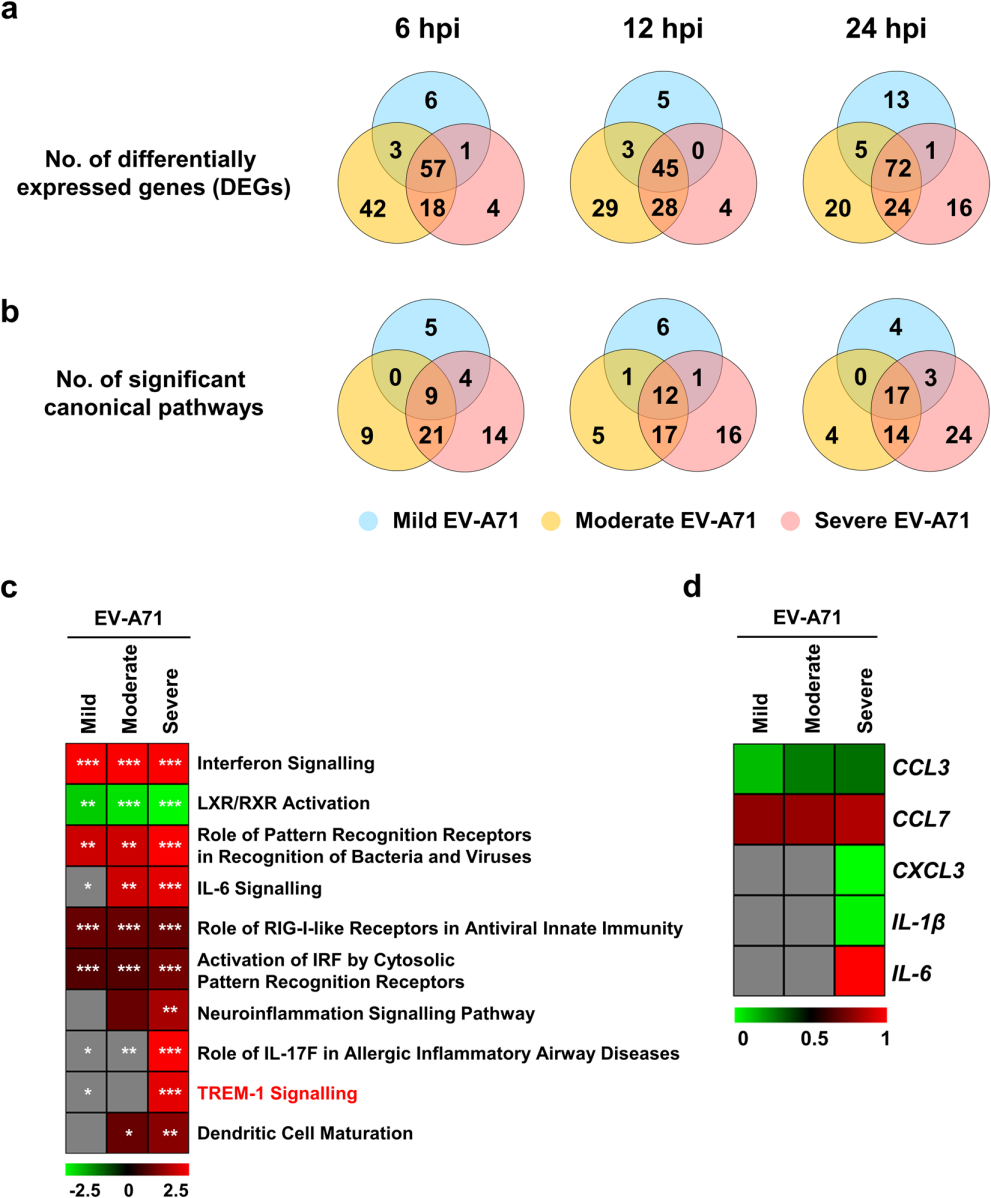
Differences in transcriptomic profiles of human PBMCs infected with different EV-A71 isolates. Primary human PBMCs (n = 4) were infected with mild, moderate, severe and heat-inactivated EV-A71 isolates at MOI 5, and harvested at 0, 6, 12 and 24 hpi for transcriptomic analysis by RNA-seq. Results of infected conditions were compared to heat-inactivated control, and the log_2_-(fold change) values were analysed in Ingenuity Pathway Analysis (IPA). **(a,b)** Venn diagrams illustrating the number of significant **(a)** differentially expressed genes (DEGs) and **(b)** canonical pathways of the different EV-A71 isolates at 6, 12 and 24 hpi. **(c)** Heat-map showing the activation *z*-scores of the top 10 canonical pathways at 24 hpi as determined by IPA. Asterisks indicate the significance of the pathway in respective isolates. **(d)** Heat-map showing normalised log_2_-(fold change) values of the genes involved in TREM-1 signalling pathway at 24 hpi. Grey boxes indicate no values. (*p < 0.05; **p < 0.01; ***p < 0.001).

Given that the different EV-A71 isolates belonged to the same sub-genotype B5 (Supplementary Fig. S1b), and yet were able to give rise to such varied clinical outcomes in patients (Fig. 1a), we then sought to understand this by identifying and comparing the unique DEGs of the EV-A71 isolates at the respective time-points of 6, 12 and 24 hpi. The three isolates showed distinctive trends (Fig. 4a). While the number of unique genes increased slightly from 6 to 13 DEGs (6 to 24 hpi) in the mild-infected condition, the moderate EV-A71-infected PBMCs displayed an opposite trend, with a decrease in the numbers from 42 to 20 DEGs (Fig. 4a). On the other hand, infection of PBMCs with severe EV-A71 isolate showed an increasing number of DEGs specific to this isolate (4 to 16 DEGs at 6 and 24 hpi) (Fig. 4a). Examples of enriched DEGs distinct to each isolate were nuclear protein 1 (*NUPR1*) for mild, chemokine (C-X-C motif) ligand 13 (*CXCL13*) for moderate, and interleukin-6 (*IL-6*) for severe EV-A71 infections (Supplementary Table S4). An analysis of the unique DEGs with IPA revealed significant canonical pathways similar to the observations as in Fig. 4a (Fig. 4b).

Since the numbers of activated canonical pathways in EV-A71-infected PBMCs were the highest at 24 hpi (Figs. 3b and 4b), the differential genes of each isolate at this particular time-point were input into IPA and comparison analyses were carried out (Supplementary Table S4). The top 10 canonical pathways, based on the activation *z*-scores as determined by IPA, were selected for further analysis (Fig. 4c and Supplementary Table S5). Remarkably, PBMCs infected with severe EV-A71 showed a very distinctive response. Pathways such as IL-6, neuroinflammation, role of IL-17F in allergic inflammatory airway diseases, and TREM-1 signalling processes were significantly and highly activated but were absent or lacking in the mild and moderate EV-A71-infected conditions (Fig. 4c). Notably, the TREM-1 signalling pathway was most distinctive in severe EV-A71 infection (Fig. 4c). The expression of genes involved in this pathway were strongly enriched for *CXCL3*, *IL-1β* and *IL-6* in the severe-infected condition (Fig. 4d). Moreover, TREM-1 pathway had not been explored before in the context of EV-A71 infection, and as such, this pathway was further studied in subsequent experiments.

### Reduced TREM-1 expression mitigates EV-A71 infection

To verify the role of TREM-1 signalling pathway as an important mediator in EV-A71 infection, a series of blocking experiments were carried out in which healthy donor PBMCs were pre-treated with 100 ng/ml of synthetic peptide LP17 (Supplementary Fig. S3a). LP17 was previously shown to be able to compete with the ligand and block the TREM-1 receptor, thus inhibiting inflammatory responses^26–28^. A scrambled form of the peptide, named sLP17, was used as negative control (Supplementary Fig. S3a). PBMCs were then infected with mild, moderate and severe EV-A71 isolates at MOI 5, as well as mock-infected, before fresh media containing the final concentration of peptides were added (Supplementary Fig. S3a). In addition, as positive controls, PBMCs were subjected to lipopolysaccharide (LPS) treatment in the presence of peptides. To validate that the blocking was efficient, total RNA samples of peptide- and LPS-treated PBMCs were subjected to gene expression studies by qRT-PCR (Supplementary Fig. S3b). LPS controls with LP17 peptide treatment resulted in decreased expression levels of the genes of interest, especially *IL-6* and *CCL7*, indicating a successful TREM-1 inhibition (Supplementary Fig. S3b).

**Figure 5:**
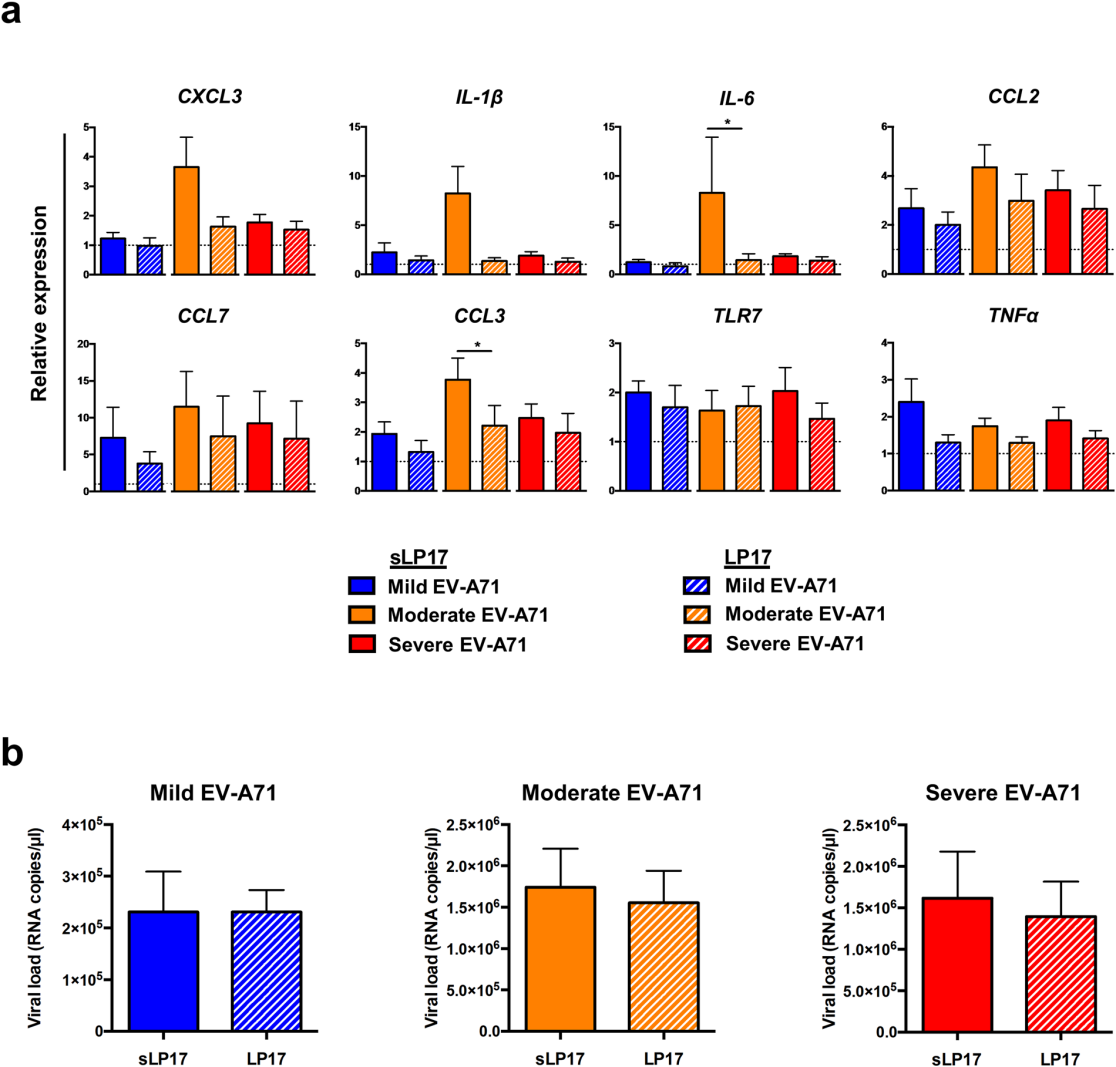
Blocking of TREM-1 by peptide LP17 decreased the expression levels of pro-inflammatory genes during moderate and severe EV-A71 infection. Human primary PBMCs (n = 7) were pre-treated with 100 ng/ml of LP17 or the control sLP17 peptide, before infection with mild, moderate, or severe EV71 isolates at MOI 5. Mock-infected PBMCs were used as negative control. Cells were replenished with fresh media containing respective peptides and harvested at 24 hpi for gene expression and viral load quantifications. **(a)** Total RNA samples were extracted and gene expression studies were performed via qRT-PCR using 10 ng/µl of RNA. Relative expression levels of genes were calculated by comparing the infected conditions to the respective mock. Bar graphs illustrating the expression levels of genes involved in the TREM-1 pathway. Dotted line indicates the baseline levels from mock. **(b)** Viral load levels were quantified by qRT-PCR of the VP1 RNA copies. Data are presented as mean ± SD. Statistical analysis was carried out with Wilcoxon matched-pairs signed rank test (*p < 0.05).

Similarly, in the EV-A71-infected conditions, a reduced gene expression profile was observed for the moderate and severe EV-A71 isolates (Fig. 5a). However, the intensity of TREM-1 inhibition was more pronounced in moderate isolate infection when compared to severe-infected condition (Fig. 5a). On the other hand, mild EV-A71 showed minimal changes in the relative level of gene expression, suggesting that TREM-1 activation in this isolate could be too low to detect for any inhibition (Fig. 5a). The impact of EV-A71 viral replication upon TREM-1 inhibition was then assessed by qRT-PCR. Consistent with the minor effects observed in the gene expression results, infection with mild EV-A71 showed no differences in the number of viral VP1 RNA copies under the blocked and control states (Fig. 5b). However, despite changes in the gene expression profile, the treatment with LP17 to inhibit TREM-1 had no significant effect on viral VP1 RNA load in both the moderate and severe EV-A71 infections (Fig. 5b).

## Discussion

Patients suffering from EV-A71 infections can present different clinical manifestations and severity. Previous studies have suggested that outcomes of EV-A71 infections could be influenced by the genetic makeup of the virus, as well as the immune status of the patient^21,29–32^. The amino acid residue at position 145 of VP1 protein varies among EV-A71 isolates, comprising of either glycine (G), glutamine (Q) or glutamic acid (E)^33^. Variability at this position is critical as it determines the attachment of the virus to the cell surface, thus influencing its virulence^33,34^. Reports have shown that isolates which have G or Q residues are isolated more frequently from patients with severe EV-A71 infections^33,35–38^. Indeed, the moderate and severe EV-A71 isolates in this study possess the Q and G residues respectively, while the mild isolate has the E residue at this position (Supplementary Fig. S4a). In addition, nucleotide alignment of the various genes of the isolates revealed that non-structural gene 3A was dissimilar between the isolates (92% identity) (Supplementary Table S1). The 3A protein has been shown to be important for virus RNA replication, with its involvement in the formation of replication organelles^39–41^. It was recently reported that recruitment of phosphatidylinositol 4-kinase IIIβ (PI4KB) by the interaction of host factor acyl-CoA binding domain containing 3 (ACBD3) and EV-A71 3A facilitated viral replication^42^. Sequence alignment of the 3A protein reveals only one amino acid difference at position 61 (Supplementary Fig. S4b) in which the mild isolate has an isoleucine (I) residue whereas the moderate and severe isolates have valine (V) instead. Despite these differences, all EV-A71 isolates were highly genetically identical overall (at least 96%). Yet, viral severity-dependent effects were observed in muscle RD cells and in PBMCs. Moderate and severe EV-A71 isolates demonstrated high levels of infection and replication, while mild EV-A71 showed lower infectivity. However, in neuronal SH-SY5Y cells, mild and moderate EV-A71 exhibited similar levels of replication kinetics as the severe isolate, suggesting that EV-A71 replication is cell-type dependent^43^ and that the mild and moderate isolates could potentially cause neuropathogenesis if the blood-brain barrier permeability is compromised. Therefore, the results support the importance of the virus’ genetic component in determining virulence and in shaping clinical outcomes in patients.

Intriguingly, between the moderate and severe EV-A71 infections in PBMCs, the latter exhibited a steady increase in infection and replication over time while the former showed a strong infection only in the initial phase. This was also supported in the transcriptomic results, in which the viral recognition pathway by PRR remained activated specifically for severe EV-A71, thus emphasising the ongoing replication of virus over time. In addition, several pathways such as neuroinflammation and IL-6 signalling were identified to be strongly associated or unique to severe EV-A71 infection. Cytokines IL-1β and IL-6 are the common denominators of these pathways and are therefore likely to play a role in the severe pathogenesis of EV-A71. In earlier reports, levels of IL-1β, IL-6 and TNFα were found to be significantly elevated in fatal EV-A71 patients with encephalitis and pulmonary oedema, when compared to patients with no complications^31,44–46^. Production of such inflammatory cytokines are mediated by monocytes, macrophages, as well as T and B lymphocytes^46^. Furthermore, these immune cells were the subsets shown to be the most susceptible to EV-A71 in this study and elsewhere^47–49^.

The link between pro-inflammatory response and severe EV-A71 outcome is also further strengthened with the identification of TREM-1 activation, which is unique and distinct to the severe isolate. TREM-1 is a cell surface receptor that is highly expressed on monocytes and neutrophils, and is known as a critical player in the amplification of inflammation via the production of pro-inflammatory mediators^50^. TREM-1 can also exist as a soluble variant, with its expression levels indirectly proportional to that of its receptor form^50^. To date, TREM-1 modulation in viral infections has been described for dengue, Marburg (MARV), Ebola (EBOV) and human immunodeficiency (HIV) viruses among others^28,51,52^. In dengue-infected patients, the expression of TREM-1 on neutrophils was significantly reduced in the acute phase of the disease, with high levels of soluble TREM-1 in sera^51^. On the other hand, HIV upregulated TREM-1 expression on human macrophages, preventing its apoptosis and mediating infection^52^. In addition, MARV and EBOV were able to activate TREM-1 on human neutrophils *in vitro*, and the blocking of TREM-1 receptor by LP17 peptide treatment resulted in diminished production of TNFα and IL-1β^28^. Although a decrease in the expression of pro-inflammatory mediators was also observed in this study, the effect of the treatment was most significant for the moderate isolate of EV-A71. Moreover, although a lower viral load was observed in the blocked conditions of moderate and severe infections, results were not significant when compared to the control condition. Therefore, inhibition of TREM-1 specifically targeted the downstream immune response after virus infection (Fig. 5). Previously, TREM-1-deficient mice infected with either *Leishmania major*, influenza virus or *Legionella pneumophila* were able to demonstrate reduced morbidity and immune-associated pathologies, with minimal effect on pathogen clearance^53^. Another plausible explanation could be attributed to the host genetic factors, as donor-to-donor variations were observed in our study. Gene polymorphisms within TREM-1 have been linked to the development of inflammation and sepsis^54^, and it has been reported that there is a positive association between patients carrying the *TREM-1* rs2234237T allele and the development of severe malaria, due to the presence of higher levels of soluble TREM-1 in the plasma of these patients^55^. Given that polymorphisms in EV-A71 receptors, scavenger receptor class B member 2 (SCARB2) and P-selectin glycoprotein ligand-1 (PSGL-1), have been shown to affect EV-A71 susceptibility and infection respectively^56^, genomic variations within TREM-1 could also play a probable role in modulating EV-A71 infection and outcomes. Therefore, further investigations in patients are required to support and validate the findings of this study.

In conclusion, this study characterised and compared the cellular responses elicited by EV-A71 isolates of differing clinical severity and revealed the potential novel involvement of TREM-1 in the pathogenesis of severe EV-A71 infection. Importantly, TREM-1 expression levels could serve as a prognostic marker for severe EV-A71 infections in patients, and might point the way towards future therapies.

## Methods

### Ethics approval

Human blood and apheresis cone bloods were obtained from healthy volunteers with written informed consent, in accordance with the Declaration of Helsinki for Human Research, and with guidelines from the Health Sciences Authority of Singapore. Study protocols were approved by the SingHealth Centralised Institutional Review Board (CIRB reference numbers: 2017/2806 and 2017/2512).

### Cells and viruses

Human rhabdomyosarcoma (RD; ATCC CCL-136) and neuroblastoma (SH-SY5Y; ATCC CRL-2266) cell lines were cultured in Dulbecco’s Modified Eagle Medium (DMEM) and Roswell Park Memorial Institute (RPMI) (GE Healthcare Life Sciences) respectively, supplemented with 10% foetal bovine serum (FBS; GE Healthcare Life Sciences) at 37 °C with 5% CO_2_. EV-A71 viral isolates used in this study were originally isolated from six patients from the 2006 outbreak in Sarawak, Malaysia (sequence data submitted to the GenBank databases under accession numbers MN053430, MN053431, MN053432, MN053433, MN053434 and MN053435). Viruses were propagated for three passages and titred in RD cells, and stored at −80 °C. The same passage number of viruses were used for infection experiments. Heat-inactivation of virus was done at 65 °C for 45 min.

### Tissue culture infective dose 50 (TCID50) assay

Viral titers were determined in RD cells via TCID50 assay. Briefly, RD cells (1.5 × 10^4^) were seeded in 96-well microtiter plates overnight before infection with serial dilutions of EV-A71 isolates in serum-free DMEM. After 1.5 h, cells were refreshed with DMEM with 10% FBS and incubated for 4 days at 37 °C. Cells were fixed with 10% formalin (Sigma-Aldrich) before being stained with 0.5% crystal violet (Sigma-Aldrich). Titre was calculated using Reed-Muench method^57^.

### Genome sequencing and phylogenetic analysis

Viral RNA of the EV-A71 isolates was extracted using QIAamp Viral RNA Mini Kit (Qiagen) as per manufacturer’s instructions. 30 ng of viral RNA samples were subjected to cDNA synthesis using Illumina TruSeq RNA sample preparation kit version 2 (Low-Throughput protocol) according to manufacturer’s protocol, except for the following modifications: 1. Skip mRNA purification and start with RNA fragmentation 2. use of 12 PCR cycles, 3. two additional round of Agencourt Ampure XP SPRI beads (Beckman Coulter) to remove >600 bp double stranded cDNA. The length distribution of the cDNA libraries was monitored using DNA 1000 kits on the Agilent bioanalyser. All samples were subjected to an indexed SE sequencing run of 1 × 51 cycles on an Illumina MiSeq (18 samples/lane). EV-A71 sub-genogroup B phylogenetic tree was generated by maximum-likelihood analysis of complete VP1 nucleotide sequences aligned using MUSCLE in MEGA. The tree was rooted to the prototype genogroup A strain. Sequences were identified by GenBank accession, year of isolation, and country of origin. Tree robustness was evaluated by bootstrap analysis using 1000 pseudo-replicate sequences. Major clades of bootstrap values of more than 75% were indicated at relevant branch nodes.

### *In vitro* infection of cell lines

RD and SH-SY5Y cells (1 × 10^6^) seeded in 60 mm dish were infected with the EV-A71 isolates prepared in serum-free media at multiplicity of infection (MOI) 10 and 1, respectively^25,58–61^. After 1.5 h, virus inoculum was removed and replaced with media containing 10% FBS. Cells were incubated at 37 °C and harvested at time-points 0, 3, 6, 12, and 24 hours post-infection (hpi). Mock-infected controls were cells without any virus infection.

### *Ex vivo* treatment and infection of human peripheral blood mononuclear cells (PBMCs)

PBMCs were first isolated from a blood apheresis cone by gradient centrifugation using Ficoll-Hypaque method as previously described^62,63^. PBMCs (2 × 10^6^) were then infected with EV-A71 viral isolates at MOI 5 in serum-free Iscove’s Modified Dulbecco’s Medium (IMDM; Gibco)^60^. After 2 h, virus inoculum was removed and replaced with IMDM with 10% human serum (HS; Innovative Research Inc). Cells were incubated at 37 °C and harvested at time-points 0, 6, 12, and 24 hpi. Mock-infected controls were cells without any virus infection, whereas heat-inactivated controls were cells infected with heat-inactivated virus. For TREM-1 blocking experiments, isolated PBMCs were first pre-treated with 100 ng/ml of LP17 peptide (LQVTDSGLYRCVIYHPP) or its scrambled form, sLP17 (TDSRCVIGLYHPPLQVY) (Pepscan Systems) as previously described^26,27^, in serum-free IMDM. After 30 min incubation at 37 °C, PBMCs were then infected with EV-A71. Subsequently, peptide-virus solutions were removed and cells were refreshed with 100 ng/ml of sLP17 or LP17 peptides in IMDM and 10% HS. Mock-infected, and LPS-treated PBMCs (50 ng/ml; Sigma-Aldrich) were used as negative and positive controls, respectively.

### Viral RNA extraction and viral RNA quantification

Viral RNA from 140 μl of culture supernatant was extracted using QIAamp Viral RNA Mini Kit (Qiagen) according to manufacturer’s instructions. Viral load quantification was performed with a VP1-targeted quantitative real-time polymerase chain reaction (qRT-PCR) adapted from Tan and colleagues^64^, with forward and reverse primers (GAGAGCTCTATAGGAGACAGT and GAGAGCTCTATAGGAGACAGT respectively), and an additional novel lab-designed FAM-labeled molecular beacon probe (ACCCACAGGTCAAAACACACA) taken from consensus sequence of the six EV-A71 isolates. RT-PCR reactions were prepared using QuantiTect Probe RT-PCR Kit (Qiagen), with final concentrations of primers and probe used were 400 nM and 200 nM respectively. The thermal cycling conditions were: 50 °C for 30 min, 95 °C for 15 min, followed by a 2-step cycle of a cycle at 94 °C for 15 min, and 45 cycles at 55 °C for 1 min on 7900HT Fast Real-Time PCR System (Applied Biosystems). Assay exclusivity of EV-A71-VP1 was confirmed by testing viral RNA extracted from dengue viruses (DENV-1 to DENV-4), Chikungunya virus (CHIKV), and O’nyong’nyong virus (ONNV). Analytical sensitivity was also determined using quantitated EV-A71 RNA transcripts. The lower limit of detection was estimated as 10 copies for the VP1 gene target. Copy numbers of EV-A71 RNA were determined using the Ribogreen RNA-specific Quantitation Kit (Invitrogen). RNA transcripts from 10 to 10^9^ copies were performed in pentaplicates to construct a standard curve to estimate EV-A71 copy number in samples.

### Flow cytometry

RD and SH-SY5Y cells from *in vitro* infections were stained with LIVE/DEAD Fixable Aqua Dead Stain (Life Technologies), fixed with 1X BD FACS Lysing Solution (BD Biosciences), and permeabilised with 1X BD FACS Permeabilising Solution 2 (BD Biosciences). Subsequently, cells were stained with EV-A71 VP1 protein specific mouse monoclonal antibody (clone 10F0; Abcam), and counter-stained with fluorophore-tagged goat anti-mouse IgG (H + L) antibody (Thermo Fisher Scientific), before being acquired with BD FACSCanto II (BD Biosciences). PBMCs from *ex vivo* infections were stained and fixed similarly as above, and permeabilised with 0.5% Triton X-100 (Sigma-Aldrich). Cell surface staining was performed with mouse anti-human CD45 (clone HI30; BioLegend), CD3 (clone SP34-2; BD Biosciences), CD19 (clone SJ25C1; eBioscience), CD14 (clone M5E2; BD Pharmingen), CD16 (clone 3G8; BioLegend), CD56 (clone AF12-7H3; Miltenyi Biotec), CD11c (clone 3.9; BioLegend), HLA-DR (clone L243; BioLegend), followed by EV-A71 VP1 staining and its counter-stain, as above. Cells were acquired with BD LSRFortessa (BD Biosciences). Flow cytometry data were analysed with FlowJo version 9.3.2 (Tree Star, Inc).

### Total RNA extraction

Total RNA from cells of PBMCs infections were extracted using RNeasy Micro Kit (Qiagen) according to manufacturer’s instructions, and the concentrations quantified with Nanodrop 1000 spectrophotometer (Thermo Fisher Scientific).

### Gene expression

Total RNA samples were diluted to a concentration of 10 ng/μl prior to gene expression studies by qRT-PCR using QuantiFast SYBR green RT-PCR kit (Qiagen) in 7900HT Fast Real-Time PCR System (Applied Biosystems)^65,66^ according to manufacturer’s protocol. The C_t_ values for *CXCL3*, *IL-1β, IL-6*, *CCL2*, *CCL7*, *CCL3*, *TLR7*, *TNFα* and *GAPDH* (housekeeping gene) were determined. The fold change relative to mock-infected samples for each gene was determined using the ΔC_t_ method and Microsoft Excel 2016^65,66^. The sequences of the primers used are in Supplementary Table S6.

### RNA-seq and differential gene expression analysis

The methods for RNA-seq and differential expression were previously described^67–69^ and are briefly described below.

### RNA-seq

RNA-seq was performed as previously described^67–69^. Briefly, RNA samples were DNase-treated using Ambion Turbo DNA-free Kit (Invitrogen), purified using Agencourt Ampure XP beads (Beckman Coulter), followed by a Ribozero treatment using the Epicentre Ribo-Zero Gold Kit (Human/Rat/Mouse) (Ilumina), and purified again with Ampure XP beads. Successful depletion was then quality tested using Qubit 4 Fluorometer (Thermo Fisher) and Agilent 2100 Bioanalyser (Agilent Technologies), and all of the depleted RNA was used as input material for the ScriptSeq v2 RNA-Seq Library Preparation protocol. Following 14 cycles of amplification, the libraries were purified using Ampure XP beads. Each library was quantified using Qubit, and the size distribution was assessed using the AATI Fragment Analyser (Advanced Analytical). Final libraries were pooled in equimolar amounts, and the quantity and quality of each pool was assessed by the Fragment Analyser and subsequently by qPCR using the Illumina Library Quantification Kit (Kapa Biosystems) on a Light Cycler LC480II (Roche) according to manufacturer’s instructions. The template DNA was denatured according to the protocol described in the Illumina cBot User guide and loaded at 12 pM concentration. To improve sequencing quality control 1% PhiX was spiked-in. Sequencing was carried out on three lanes of an Illumina HiSeq. 2500 with version 4 chemistry, generating 2 × 125 bp paired-end reads.

### Bioinformatics analysis

Briefly, base calling and de-multiplexing of indexed reads were performed by CASAVA version 1.8.2 (Illumina) to produce 30 samples from the 5 lanes of sequence data, in fastq format. The raw fastq files were trimmed to remove Illumina adapter sequences using Cutadapt version 1.2.1^70^. The option “-O 3” was set, so that the 3′ end of any reads which matched the adapter sequence over at least 3 bp was removed. The reads were further trimmed to remove low quality bases, using Sickle version 1.200 with a minimum window quality score of 20. After trimming, reads shorter than 50 bp were removed. If both reads from a pair passed this filter, each was included in the R1 (forward reads) or R2 (reverse reads) file. If only one of a read pair passed this filter, it was included in the R0 (unpaired reads) file. The reference genome used for alignment was the human reference genome assembly GRCh38. The reference annotation was downloaded from the Ensembl ftp site (ftp://ftp.ensembl.org/pub/release-77/gtf/homo_sapiens/Homo_sapiens.GRCh38.77.gtf.gz). The annotated file contained 63,152 genes. R1/R2 read pairs were mapped to the reference sequence using TopHat2 version 2.1.0^71^ which calls the mapper Bowtie2 version 2.0.10^72^. Data are accessible at NCBI’s Gene Expression Omnibus (GEO) database (accession number GSE135964).

### Differential gene expression and functional analysis

Mapped reads were further analysed using edgeR version 3.3^73^ to calculate normalised counts per million (CPM), to identify genes differentially expressed between infected and heat-inactivated conditions. The log_2_-(fold change) values of such genes were uploaded into Ingenuity Pathway Analysis (IPA; Qiagen) and subsequently used for gene ontology and pathway analysis. IPA was performed to identify canonical pathways, and comparison analysis was used to determine the most significant pathways across the different isolates and time-points. Activation *z*-scores and *p*-values associated with the identified functional pathways were determined by IPA. Heat-maps were generated using TM4 Multi-Experiment Viewer^74^.

### Statistical analysis

All statistical analyses were performed using GraphPad Prism 7 (GraphPad Software). For cell lines and PBMCs infection experiments, Kruskal-Wallis with Dunn’s multiple comparison test was used to compare the EV-A71 isolates at each time-point. For peptide-PBMCs experiments, Wilcoxon matched-pairs signed rank test was used in the comparison of LP17 and control sLP17 peptides in the respective infected condition. *P* values less than 0.05 are considered to be statistically significant.

## Supplementary information

Supplementary information.

## References

[1] McMinn PC (2002). An overview of the evolution of enterovirus 71 and its clinical and public health significance. FEMS Microbiol. Rev..

[2] Ooi MH, Wong SC, Lewthwaite P, Cardosa MJ, Solomon T (2010). Clinical features, diagnosis, and management of enterovirus 71. Lancet Neurol..

[3] Liu MY (2016). Characterization of enterovirus 71 infection and associated outbreak of Hand, Foot, and Mouth Disease in Shawo of China in 2012. Sci. Rep..

[4] Bruu, A.-L. Enteroviruses: Polioviruses, Coxsackieviruses, Echoviruses and Newer Enteroviruses. *A Practical Guide to Clinical Virology*, 10.1002/0470857285.ch6 (2003).

[5] Esposito S, Principi N (2018). Hand, foot and mouth disease: Current knowledge on clinical manifestations, epidemiology, aetiology and prevention. Eur. J. Clin. Microbiol. Infect. Dis..

[6] Crabol Y (2017). A prospective, comparative study of severe neurological and uncomplicated hand, foot and mouth forms of paediatric enterovirus 71 infections. Int. J. Infect. Dis..

[7] Cox JA, Hiscox JA, Solomon T, Ooi MH, Ng LFP (2017). Immunopathogenesis and virus-host interactions of enterovirus 71 in patients with hand, foot and mouth disease. Front. Microbiol..

[8] Wang Q (2014). Clinical features of severe cases of hand, foot and mouth disease with EV71 virus infection in China. Arch. Med. Sci..

[9] Brown BA, Pallansch MA (1995). Complete nucleotide sequence of enterovirus 71 is distinct from poliovirus. Virus Res..

[10] Bessaud M (2014). Molecular comparison and evolutionary analyses of VP1 nucleotide sequences of new African human enterovirus 71 isolates reveal a wide genetic diversity. PLoS One.

[11] Schmidt NJ, Lennette EH, Ho HH (1974). An apparently new enterovirus isolated from patients with disease of the central nervous system. J. Infect. Dis..

[12] Chang PC, Chen SC, Chen KT (2016). The current status of the disease caused by enterovirus 71 infections: Epidemiology, pathogenesis, molecular epidemiology, and vaccine development. Int. J. Environ. Res. Public Health.

[13] Tee KK (2010). Evolutionary genetics of human enterovirus 71: Origin, population dynamics, natural selection, and seasonal periodicity of the VP1 gene. J. Virol..

[14] Prager P, Nolan M, Andrews IP, Williams GD (2003). Neurogenic pulmonary edema in enterovirus 71 encephalitis is not uniformly fatal but causes severe morbidity in survivors. Pediatr. Crit. Care Med..

[15] Ho M (1999). An epidemic of Enterovirus 71 infection in Taiwan. N. Engl. J. Med..

[16] Chan LG (2000). Deaths of children during an outbreak of hand, foot, and mouth disease in Sarawak, Malaysia: Clinical and pathological characteristics of the disease. Clin. Infect. Dis..

[17] Chan KP (2003). Epidemic hand, foot and mouth disease caused by human enterovirus 71, Singapore. Emerg. Infect. Dis..

[18] Kuo RL, Shih SR (2013). Strategies to develop antivirals against enterovirus 71. Virol. J..

[19] Li J-X (2016). Two-year efficacy and immunogenicity of Sinovac Enterovirus 71 vaccine against hand, foot and mouth disease in children. Expert Rev. Vaccines.

[20] Yi E-J, Shin Y-J, Kim J-H, Kim T-G, Chang S-Y (2017). Enterovirus 71 infection and vaccines. Clin Exp Vaccine Res.

[21] Ooi MH (2007). Human enterovirus 71 disease in Sarawak, Malaysia: A prospective clinical, virological, and molecular epidemiological study. Clin. Infect. Dis..

[22] Ooi MH (2009). Identification and validation of clinical predictors for the risk of neurological involvement in children with hand, foot, and mouth disease in Sarawak. BMC Infect. Dis..

[23] Pérez-Ruiz M, Navarro-Marí JM, del Valle E, Rosa-Fraile M (2003). Human rhabdomyosarcoma cells for rapid detection of enteroviruses by shell-vial assay. J. Med. Microbiol..

[24] La Monica N, Racaniello VR (1989). Differences in replication of attenuated and neurovirulent polioviruses in human neuroblastoma cell line SH-SY5Y. J. Virol..

[25] Xu L-J (2013). Global transcriptomic analysis of human neuroblastoma cells in response to enterovirus type 71 infection. Plos One.

[26] Gibot S (2004). A soluble form of the triggering receptor expressed on myeloid cells-1 modulates the inflammatory response in murine sepsis. J. Exp. Med..

[27] Buonsanti C (2006). Modulation of the triggering receptor expressed on the myeloid cell type 1 pathway in murine septic shock. Infect. Immun..

[28] Mohamadzadeh M (2006). Activation of triggering receptor expressed on myeloid cells-1 on human neutrophils by Marburg and Ebola Viruses. J. Virol..

[29] Huang CC (1999). Neurologic complications in children with enterovirus 71 infection. N. Engl. J. Med..

[30] Ho M (2000). Enterovirus 71: the virus, its infections and outbreaks. J. Microbiol. Immunol. Infect..

[31] Griffiths MJ (2012). In enterovirus 71 encephalitis with cardio-respiratory compromise, elevated interleukin 1beta, interleukin 1 receptor antagonist, and granulocyte colony-stimulating factor levels are markers of poor prognosis. J. Infect. Dis..

[32] Li B (2017). A novel enterovirus 71 (EV71) virulence determinant: The 69th residue of 3C protease modulates pathogenicity. Front. Cell. Infect. Microbiol..

[33] Fujii K (2018). VP1 amino acid residue 145 of enterovirus 71 is a key residue for its receptor attachment and resistance to neutralizing antibody during cynomolgus monkey infection. J. Virol..

[34] Nishimura Y (2013). Enterovirus 71 binding to PSGL-1 on leukocytes: VP1-145 acts as a molecular switch to control receptor interaction. Plos Pathog..

[35] Li R, Zou Q, Chen L, Zhang H, Wang Y (2011). Molecular analysis of virulent determinants of enterovirus 71. Plos One.

[36] Chang S-C (2012). Genetic characterization of enterovirus 71 isolated from patients with severe disease by comparative analysis of complete genomes. J. Med. Virol..

[37] Liu Y (2014). A novel finding for enterovirus virulence from the capsid protein VP1 of EV71 circulating in mainland China. Virus Genes.

[38] Zhang B (2014). The variations of VP1 protein might be associated with nervous system symptoms caused by enterovirus 71 infection. BMC Infect. Dis..

[39] Arita M, Takebe Y, Wakita T, Shimizu H (2010). A bifunctional anti-enterovirus compound that inhibits replication and the early stage of enterovirus 71 infection. J. Gen. Virol..

[40] Gao Q (2015). Discovery of itraconazole with broad-spectrum *in vitro* antienterovirus activity that targets nonstructural protein 3A. Antimicrob. Agents Chemother..

[41] Ishikawa-Sasaki K, Sasaki J, Taniguchi K (2014). A complex comprising phosphatidylinositol 4-kinase IIIbeta, ACBD3, and Aichi virus proteins enhances phosphatidylinositol 4-phosphate synthesis and is critical for formation of the viral replication complex. J. Virol..

[42] Xiao X (2017). Enterovirus 3A facilitates viral replication by promoting phosphatidylinositol 4-kinase IIIβ–ACBD3 interaction. J. Virol..

[43] Too IHK (2016). Enterovirus 71 infection of motor neuron-like NSC-34 cells undergoes a non-lytic exit pathway. Sci. Rep..

[44] Lin TY (2002). Different proinflammatory reactions in fatal and non-fatal enterovirus 71 infections: implications for early recognition and therapy. Acta Paediatr..

[45] Lin T-Y, Hsia S-H, Huang Y-C, Wu C-T, Chang L-Y (2003). Proinflammatory cytokine reactions in enterovirus 71 infections of the central nervous system. Clin. Infect. Dis..

[46] Wang SM, Lei HY, Liu CC (2012). Cytokine immunopathogenesis of enterovirus 71 brain stem encephalitis. Clin. Dev. Immunol..

[47] Wang S (2010). Enterovirus 71 infection of monocytes with antibody-dependent enhancement. Clin. Vaccine Immunol..

[48] Wang J (2013). EV71-infected CD14 1 cells modulate the immune activity of T lymphocytes in rhesus monkeys..

[49] Chen L, Yeh T (2009). Enterovirus 71 infection of human immune cells induces the production of proinflammatory cytokines. Cell.

[50] Roe K, Gibot S, Verma S (2014). Triggering receptor expressed on myeloid cells-1 (TREM-1): A new player in antiviral immunity?. Front. Microbiol..

[51] Ruiz-Pacheco JA (2014). TREM-1 modulation during early stages of dengue virus infection. Immunol. Lett..

[52] Campbell GR, To RK, Spector SA (2019). TREM-1 protects HIV-1-infected macrophages from apoptosis through maintenance of mitochondrial function. MBio.

[53] Weber B (2014). TREM-1 deficiency can attenuate disease severity without affecting pathogen clearance. Plos Pathog..

[54] Aldasoro Arguinano A-A (2017). TREM-1 SNP rs2234246 regulates TREM-1 protein and mRNA levels and is associated with plasma levels of L-selectin. Plos One.

[55] Adukpo S (2016). Triggering receptor expressed on myeloid cells 1 (TREM-1) and cytokine gene variants in complicated and uncomplicated malaria. Trop. Med. Int. Heal..

[56] Yen T, Shih W, Huang Y, Lee J, Huang L (2018). Polymorphisms in enterovirus 71 receptors associated with susceptibility and clinical severity. Plos One.

[57] Reed LJ, Muench H (1938). A simple method of estimating fifty per cent endpoints. Am. J. Epidemiol..

[58] Lu J (2011). Viral kinetics of Enterovirus 71 in human habdomyosarcoma cells. World J. Gastroenterol..

[59] Lu J (2012). Enterovirus 71 disrupts interferon signaling by reducing the level of interferon receptor 1. J. Virol..

[60] Chen T-C, Lai Y-K, Yu C-K, Juang J-L (2007). Enterovirus 71 triggering of neuronal apoptosis through activation of Abl-Cdk5 signalling. Cell. Microbiol..

[61] Du X (2015). Enterovirus 71 induces apoptosis of SH-SY5Y human neuroblastoma cells through stimulation of endogenous microRNA let-7b expression. Mol. Med. Rep..

[62] Her Z (2010). Active infection of human blood monocytes by chikungunya virus triggers an innate immune response. J. Immunol..

[63] Lum FM (2017). Zika virus infects human fetal brain microglia and induces inflammation. Clin. Infect. Dis..

[64] Tan EL (2008). Rapid detection of enterovirus 71 by real-time TaqMan RT-PCR. J. Clin. Virol..

[65] Teng TS (2012). Viperin restricts chikungunya virus replication and pathology. J. Clin. Invest..

[66] Teo T-H (2015). Caribbean and La Réunion chikungunya virus isolates differ in their capacity to induce proinflammatory Th1 and NK cell responses and acute joint pathology. J. Virol..

[67] Liu X (2017). Transcriptomic signatures differentiate survival from fatal outcomes in humans infected with Ebola virus. Genome Biol..

[68] Bosworth A (2017). A comparison of host gene expression signatures associated with infection *in vitro* by the Makona and Ecran (Mayinga) variants of Ebola virus. Sci. Rep..

[69] Lum F-M (2018). Zika virus infection preferentially counterbalances human peripheral monocyte and/or NK cell activity. mSphere.

[70] Tang H (2016). Zika virus infects human cortical neural progenitors and attenuates their growth. Cell Stem Cell.

[71] Kim D (2013). TopHat2: accurate alignment of transcriptomes in the presence of insertions, deletions and gene fusions. Genome Biol..

[72] Langmead B, Salzberg SL (2012). Fast gapped-read alignment with Bowtie 2. Nat. Methods.

[73] Robinson MD, McCarthy DJ, Smyth GK (2010). edgeR: a Bioconductor package for differential expression analysis of digital gene expression data. Bioinformatics.

[74] Saeed AI (2003). TM4: a free, open-source system for microarray data management and analysis. Biotechniques.

