# Supplementary material for: TREM-1 activation is a potential key regulator in driving severe pathogenesis of enterovirus A71 infection
Source: Sci Rep. 2020 Mar 2;10:3810. doi: 10.1038/s41598-020-60761-5 (PMC7052206; doi:10.1038/s41598-020-60761-5)
Supplement: Supplementary file 1 — Supplementary information. [file 41598_2020_60761_MOESM1_ESM.pdf]

Supplementary information

TREM-1 activation is a potential key regulator in driving severe pathogenesis of enterovirus A71 infection

Siti Naqiah Amrun[1], Jeslin J.L. Tan[1], Natasha Y. Rickett[2,3], Jonathan A. Cox[1,3], Bernett Lee[1], Michael J. Griffiths[3], Tom Solomon[2,3,4], David Perera[5], Mong How Ooi[5,6], Julian A. Hiscox[1,2,3*], Lisa F.P. Ng[1,2,3,7*]

[1]Singapore Immunology Network, Agency for Science, Technology and Research, Singapore
[2]National Institute of Health Research, Health Protection Research Unit in Emerging and Zoonotic Infections, University of Liverpool, Liverpool, United Kingdom
[3]Institute of Infection and Global Health, University of Liverpool, Liverpool, United Kingdom
[4]Walton Centre NHS Foundation Trust, Liverpool Health Partners, Liverpool, United Kingdom
[5]Institute of Health and Community Medicine, University Malaysia Sarawak, Sarawak, Malaysia
[6]Department of Paediatrics, Sibu Hospital, Sibu, Sarawak, Malaysia
[7]Department of Biochemistry, Yong Loo Lin School of Medicine, National University of Singapore, Singapore

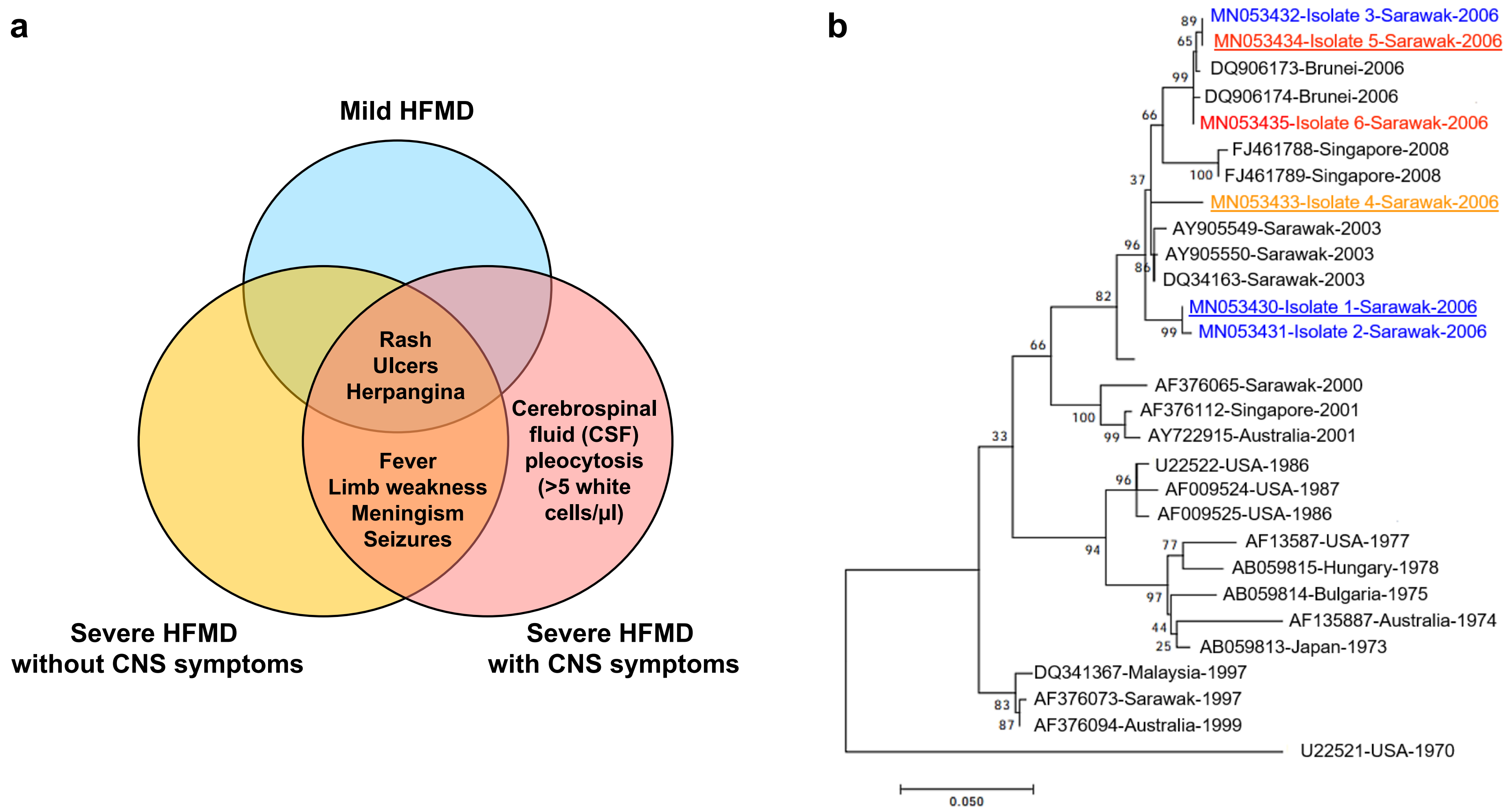

Supplementary Figure S1. Patient clinical symptoms and phylogenetic classification of EV-A71 isolates from an outbreak in Sarawak in 2006. (a) Symptomatic classification of EV-A71 patients. **(b)** EV-A71 sub-genogroup B phylogenetic tree generated by maximum-likelihood analysis of complete VP1 nucleotide sequences aligned using MUSCLE in MEGA. The tree was rooted to the prototype genogroup A strain. Sequences are identified by GenBank accession, country of origin and year of isolation. Viruses in this study are coloured according to their disease severity, and underlined are viruses that were used for characterisation. The robustness of the tree was evaluated by bootstrap analysis using 1000 pseudo-replicate sequences. Bootstrap values >75% of major clades are indicated at relevant branch nodes. All branch lengths are drawn to scale and a measurement of relative phylogenetic distance is provided by the scale at the bottom of the tree.

Supp. Figure S1 Amrun *et al.*, 2020

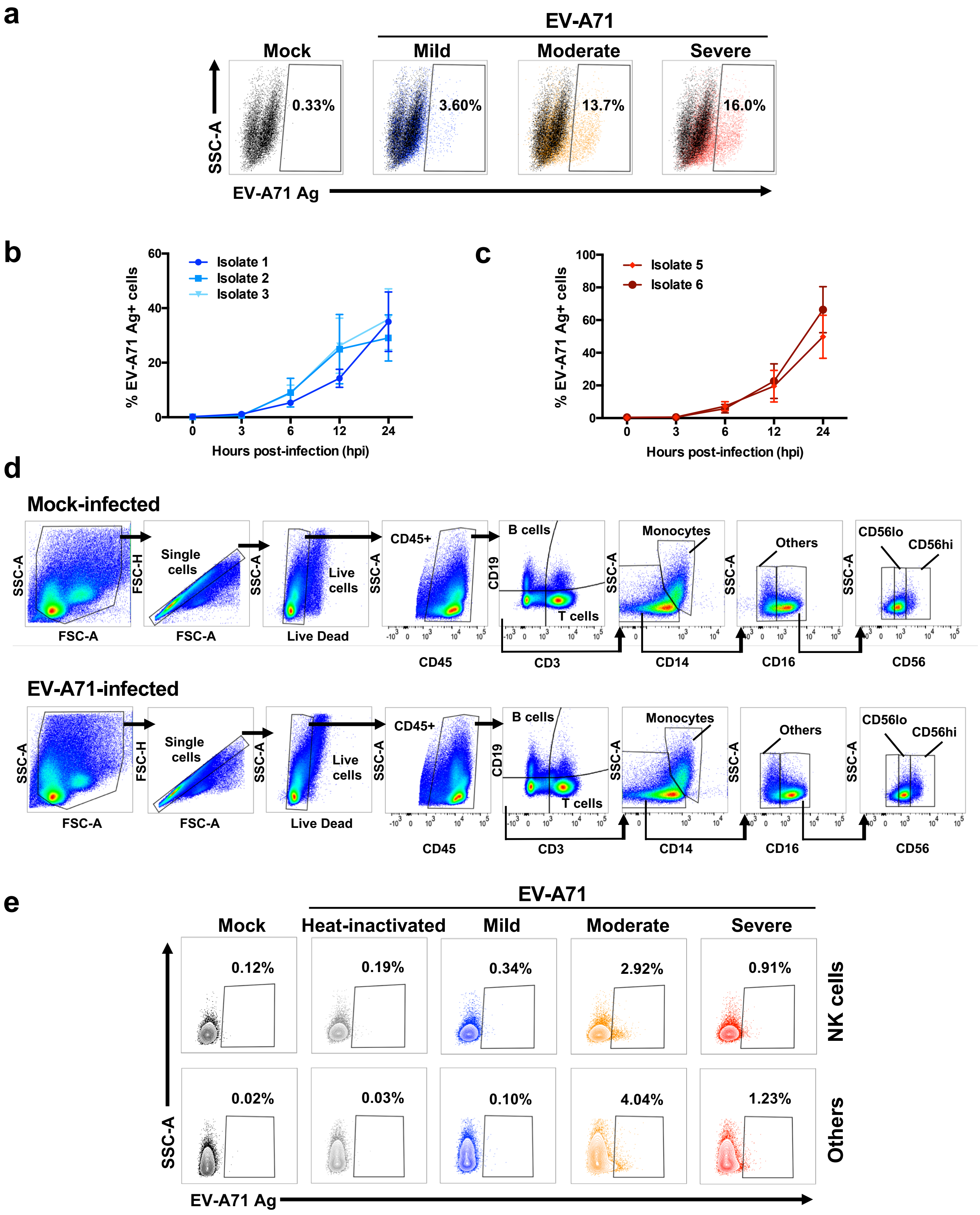

Supplementary Figure S2. EV-A71 infection in RD cells and primary human PBMCs. (a) RD cells were infected with mild (isolate 1), moderate (isolate 4) and severe (isolate 5) EV-A71 at MOI 10 and harvested at 0, 3, 6, 12 and 24 hpi. Representative dot plots of EV-A71-infected RD cells at 12 hpi. **(b-c)** Quantification of VP1 antigen by flow cytometry of EV-A71 **(b)** isolates 1, 2 and 3, and **(c)** isolates 5 and 6 in RD cells at 0, 3, 6, 12 and 24 hpi at MOI 10. Data are presented as mean ± SEM and representative of three independent experiments. Statistical analysis was carried out with Kruskal-Wallis with Dunn's multiple comparisons test to compare among EV-A71 isolates at the respective time-points. **(d-e)** Human primary PBMCs were infected with mild, moderate, severe and heat-inactivated EV-A71 isolates at MOI 5 and harvested at 0, 6, 12 and 24 hpi. **(d)** Representative illustration of flow cytometry gating strategy from one donor at 6 hpi. **(e)** Representative contour plots of EV-A71-infected cell subsets at 12 hpi.

Supp. Figure S2 Amrun *et al.*, 2020

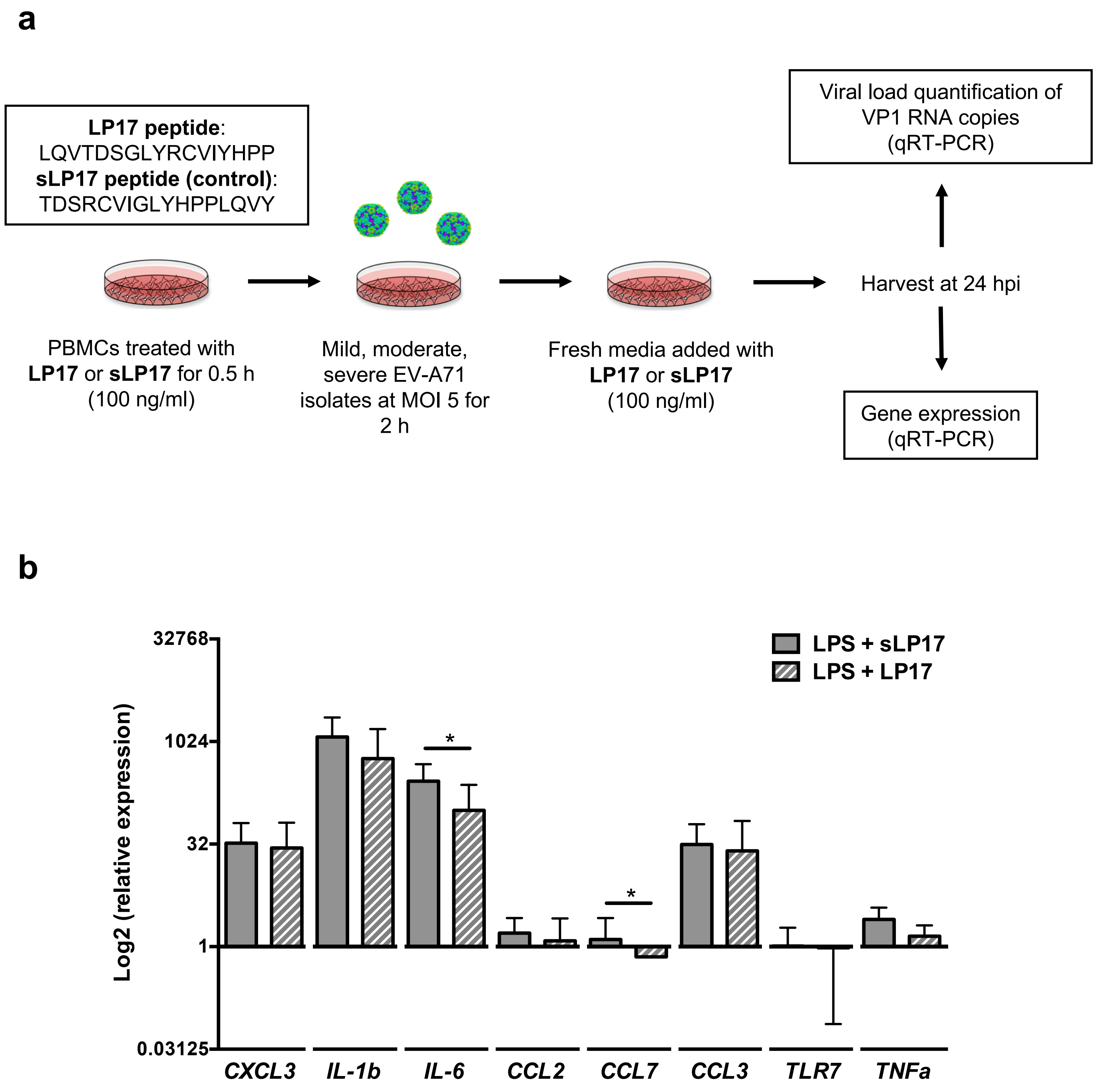

Supplementary Figure S3. Treatment of PBMCs with LP17 or sLP17 peptide. Human primary PBMCs (n=7) were pre-treated with 100 ng/ml of LP17 or the control sLP17 peptide, before infection with mild, moderate, or severe EV-A71 isolates at MOI 5. Mock-infected and LPS-treated (50 ng/ml) PBMCs were used as negative and positive controls respectively. Cells were replenished with fresh media containing respective peptides and harvested at 24 hpi for gene expression and viral load quantifications. **(a)** Schematic illustration of the method. **(b)** Bar graphs showing the relative expression levels of genes involved in the TREM-1 pathway by qRT-PCR in the different peptide treatments of LPS-treated PBMCs. Data are presented as mean ± SD. Statistical analysis was carried out with Wilcoxon matched-pairs signed rank test (*$p<0.05$).

Supp. Figure S3 Amrun *et al.*, 2020

a

```
SeverеVP1      GDRVADVIESSIGDSVSRALTQALPAPTGQNTQVSSHRLDTGEVPALQAAEIGASSNTSD   60
MildVP1        GDRVADVIESSIGDSVSRALTQALPAPTGQNTQVSSHRLDTGEVPALQAAEIGASSNTSD   60
ModerateVP1    GDRVADVIESSIGDSVSRALTQALPAPTGQNTQVSSHRLDTGEVPALQAAEIGASSNTSD   60
               ************************************************************

SevereVP1      ESMIETRCVLNSHSTAETTLDSFFSRAGLVGEIDLPLEGTTNPNGYANWDIDITGYAQMR   120
MildVP1        ESMIETRCVLNSHSTAETTLDSFFSRAGLVGEIDLPLEGTTNPNGYANWDIDITGYAQMR   120
ModerateVP1    ESMIETRCVLNSHSTAETTLDSFFSRAGLVGEIDLPLEGTTNPNGYANWDIDITGYAQMR   120
               ************************************************************

SevereVP1      RKVELFTYMRFDAEFTFVACTPTGGVVPQLLQYMFVPPGAPKPDSRESFAWQTATNPSVF   180
MildVP1        RKVELFTYMRFDAEFTFVACTPTGEVVPQLLQYMFVPPGAPKPDSRESLAWQTATNPSVF   180
ModerateVP1    RKVELFTYMRFDAEFTFVACTPTGQVVPQLLQYMFVPPGAPKPDSRESLAWQTATNPSVF   180
               ************************ ***********************:***********

SevereVP1      VKLTDPPAQVSVPFMSPASAYQWFYDGYPTFGEHKQEKDLEYGACPNNMMGTFSVRTVGS   240
MildVP1        VKLTDPPAQVSVPFMSPASAYQWFYDGYPTFGEHKQEKDLEYGACPNNMMGTFSVRTVGS   240
ModerateVP1    VKLTDPPAQVSVPFMSPASAYQWFYDGYPTFGEHKQEKDLEYGACPNNMMGTFSVRTVGS   240
               ************************************************************

SevereVP1      SKSKYPLVVRIYMRMKHVRAWIPRPMRNQNYLFKANPNYAGNSIKPTGTSRTAITTL      297
MildVP1        SKSKYPLVIRIYMRMKHVRAWIPRPMRNQNYLFKANPNYAGNSIKPTGTSRTAITTL      297
ModerateVP1    SKSKYPLVVRIYMRMKHVRAWIPRPMRNQNYLFKANPNYAGNSIKPTGTSRTAITTL      297
               ********:************************************************
```

b

```
Mild3A         GPPKFRPIRISLEEKPAPDAISDLLASVDSEEVRQYCREQGWIIPETPTNVERHLNRAVL   60
Moderate3A     GPPKFRPIRISLEEKPAPDAISDLLASVDSEEVRQYCREQGWIIPETPTNVERHLNRAVL   60
Severe3A       GPPKFRPIRISLEEKPAPDAISDLLASVDSEEVRQYCREQGWIIPETPTNVERHLNRAVL   60
               ************************************************************

Mild3A         IMQSIATVVAVVSLVYVIYKLFAGFQ   86
Moderate3A     VMQSIATVVAVVSLVYVIYKLFAGFQ   86
Severe3A       VMQSIATVVAVVSLVYVIYKLFAGFQ   86
               :*************************
```

Supplementary Figure S4. Sequence alignment of VP1 and 3A proteins. Sequence of **(a)** VP1 and **(b)** 3A proteins of mild, moderate and severe EV-A71 isolates were aligned using Clustal Omega. Boxes in red highlight the amino acid residue differences at positions **(a)** 145 in VP1 and **(b)** 61 in 3A proteins.

Supp. Figure S4 Amrun *et al.*, 2020

Supplementary Table S1: Percentage identity matrix of EV-A71 isolates

Gene	Percentage identity (%)[a]						
CDS	**Isolate**	**1**	**2**	**3**	**4**	**5**	**6**
	**1**	100	99.41	96.44	96.41	96.46	96.41
	**2**	99.41	100	96.35	96.4	96.37	96.32
	**3**	96.44	96.35	100	96.55	99.98	99.64
	**4**	96.41	96.4	96.55	100	96.56	96.53
	**5**	96.46	96.37	99.98	96.56	100	99.65
	**6**	96.41	96.32	99.64	96.53	99.65	100
VP4	**Isolate**	**1**	**2**	**3**	**4**	**5**	**6**
	**1**	100	100	96.14	95.65	96.14	95.65
	**2**	100	100	96.14	95.65	96.14	95.65
	**3**	96.14	96.14	100	96.14	100	99.52
	**4**	95.65	95.65	96.14	100	96.14	95.65
	**5**	96.14	96.14	100	96.14	100	99.52
	**6**	95.65	95.65	99.52	95.65	99.52	100
VP2	**Isolate**	**1**	**2**	**3**	**4**	**5**	**6**
	**1**	100	98.95	95.67	97.11	95.67	96.19
	**2**	98.95	100	95.67	97.38	95.67	96.19
	**3**	95.67	95.67	100	96.19	100	99.48
	**4**	97.11	97.38	96.19	100	96.19	96.72
	**5**	95.67	95.67	100	96.19	100	99.48
	**6**	96.19	96.19	99.48	96.72	99.48	100
VP3	**Isolate**	**1**	**2**	**3**	**4**	**5**	**6**
	**1**	100	98.9	96.42	96.42	96.42	96.28
	**2**	98.9	100	96.14	96.42	96.14	96.01
	**3**	96.42	96.14	100	96.83	100	99.59
	**4**	96.42	96.42	96.83	100	96.83	96.42
	**5**	96.42	96.14	100	96.83	100	99.59
	**6**	96.28	96.01	99.59	96.42	99.59	100
VP1	**Isolate**	**1**	**2**	**3**	**4**	**5**	**6**
	**1**	100	99.66	96.97	96.75	96.97	97.31
	**2**	99.66	100	96.63	96.63	96.63	96.97
	**3**	96.97	96.63	100	96.18	100	99.66
	**4**	96.75	96.63	96.18	100	96.18	96.52
	**5**	96.97	96.63	100	96.18	100	99.66
	**6**	97.31	96.97	99.66	96.52	99.66	100
2A	**Isolate**	**1**	**2**	**3**	**4**	**5**	**6**
	**1**	100	98.89	96.22	96.44	96.22	96.22
	**2**	98.89	100	95.78	95.78	95.78	95.78
	**3**	96.22	95.78	100	97.11	100	100
	**4**	96.44	95.78	97.11	100	97.11	97.11
	**5**	96.22	95.78	100	97.11	100	100
	**6**	96.22	95.78	100	97.11	100	100
	**Isolate**	**1**	**2**	**3**	**4**	**5**	**6**
	**1**	100	99.33	94.61	95.29	94.61	94.28

2B	2	99.33	100	95.29	95.96	95.29	94.95
	3	94.61	95.29	100	94.95	100	99.66
	4	95.29	95.96	94.95	100	94.95	94.61
	5	94.61	95.29	100	94.95	100	99.66
	6	94.28	94.95	99.66	94.61	99.66	100
2C	**Isolate**	**1**	**2**	**3**	**4**	**5**	**6**
	**1**	100	99.59	97.06	96.86	97.16	96.86
	**2**	99.59	100	97.06	96.86	97.16	96.86
	**3**	97.06	97.06	100	96.76	99.9	99.39
	**4**	96.86	96.86	96.76	100	96.86	96.66
	**5**	97.16	97.16	99.9	96.86	100	99.49
	**6**	96.86	96.86	99.39	96.66	99.49	100
3A	**Isolate**	**1**	**2**	**3**	**4**	**5**	**6**
	**1**	100	100	95.83	92.26	95.83	95.83
	**2**	100	100	95.83	92.26	95.83	95.83
	**3**	95.83	95.83	100	95.24	100	100
	**4**	92.26	92.26	95.24	100	95.24	95.24
	**5**	95.83	95.83	100	95.24	100	100
	**6**	95.83	95.83	100	95.24	100	100
3B	**Isolate**	**1**	**2**	**3**	**4**	**5**	**6**
	**1**	100	99.36	97.44	97.44	97.44	97.44
	**2**	99.36	100	98.08	98.08	98.08	98.08
	**3**	97.44	98.08	100	97.44	100	100
	**4**	97.44	98.08	97.44	100	97.44	97.44
	**5**	97.44	98.08	100	97.44	100	100
	**6**	97.44	98.08	100	97.44	100	100
3C	**Isolate**	**1**	**2**	**3**	**4**	**5**	**6**
	**1**	100	99.64	95.45	95.99	95.45	95.63
	**2**	99.64	100	95.45	95.99	95.45	95.63
	**3**	95.45	95.45	100	95.63	100	99.82
	**4**	95.99	95.99	97.63	100	97.63	97.81
	**5**	95.45	95.45	100	95.63	100	99.82
	**6**	95.63	95.63	99.82	97.81	99.82	100
3D	**Isolate**	**1**	**2**	**3**	**4**	**5**	**6**
	**1**	100	100	96.97	96.39	96.97	96.61
	**2**	99.57	100	96.83	96.25	96.83	96.46
	**3**	96.97	96.83	100	96.54	100	99.64
	**4**	96.39	96.25	96.54	100	96.54	96.32
	**5**	96.97	96.83	100	96.54	100	99.64
	**6**	100	96.46	99.64	96.32	99.64	100

[a]Percentage identity matrix of EV-A71 isolates based on the nucleotide sequences as determined by MUSCLE. Numbers highlighted in red are the lowest percentages in the respective regions.

Supplementary Table S2: List of common significant differentially expressed genes (DEGs) in EV-A71-infected PBMCs across time

Gene	Log$_2$-(fold change of infected over heat-inactivated)								
	Mild EV-A71			Moderate EV-A71			Severe EV-A71		
	6 hpi	12 hpi	24 hpi	6 hpi	12 hpi	24 hpi	6 hpi	12 hpi	24 hpi
APOBEC3A	1.555	2.558	3.195	2.649	3.399	3.890	2.116	3.146	3.410
AXL	1.603	2.021	1.385	2.470	2.913	1.244	1.905	2.758	1.285
DDX58	1.754	1.576	1.860	2.167	2.193	1.934	1.873	1.912	1.855
DHX58	1.519	1.492	1.645	1.843	1.892	1.661	1.634	1.813	1.657
EIF2AK2	2.178	1.947	1.751	2.519	2.245	1.927	2.280	2.102	1.926
GMPR	1.770	2.384	2.602	2.281	3.035	2.309	1.635	2.826	2.224
HELZ2	1.853	1.835	2.036	2.164	2.201	2.311	1.862	2.255	2.300
HERC5	2.870	2.562	2.361	3.106	3.270	2.006	2.871	3.003	2.033
HERC6	2.489	2.089	2.358	2.724	2.624	2.512	2.450	2.447	2.534
IFI27	1.557	2.789	2.644	3.061	3.630	3.161	2.339	3.402	2.957
IFI35	1.175	1.385	1.530	1.601	1.792	1.479	1.286	1.556	1.351
IFI44	2.442	2.475	2.315	2.629	2.864	2.293	2.423	2.706	2.234
IFI44L	2.666	2.599	2.582	2.933	3.009	2.653	2.642	2.898	2.640
IFI6	2.632	2.779	2.314	2.963	3.286	2.355	2.431	2.922	2.094
IFIH1	1.281	1.425	1.560	1.766	1.864	1.619	1.534	1.706	1.546
IFIT5	1.634	1.462	1.445	1.869	1.976	1.487	1.761	1.796	1.512
IFITM1	1.632	1.420	2.207	1.983	2.145	2.317	1.937	2.019	2.317
IFITM3	1.424	2.174	1.849	2.199	2.787	1.876	1.767	2.538	1.755
IRF7	1.401	1.661	1.701	1.707	1.903	1.741	1.465	1.784	1.742
ISG15	2.654	3.075	3.472	3.411	3.928	3.134	2.978	3.573	3.058
ISG20	1.737	1.587	2.368	2.063	2.184	2.186	1.736	1.997	2.140
LY6E	1.903	1.992	1.801	2.209	2.434	1.884	1.915	2.178	1.747
MX1	3.061	2.640	2.265	3.517	3.297	2.326	3.207	3.106	2.242
MX2	2.870	2.419	2.145	3.380	2.938	2.321	3.129	2.840	2.248
NT5C3A	1.732	1.457	2.173	2.057	2.255	2.012	1.613	1.760	2.002
OASL	1.870	2.073	2.331	2.278	2.726	2.529	1.936	2.373	2.433
PLSCR1	1.786	1.727	1.828	2.152	2.167	1.849	1.906	1.980	1.746
PNPT1	1.943	1.573	1.718	2.183	2.285	1.578	2.079	2.027	1.600
RIN2	1.078	1.258	1.145	1.850	1.865	1.553	1.461	1.650	1.231
RSAD2	3.025	3.282	3.670	3.721	4.371	3.778	3.219	3.936	3.653
RTP4	1.378	1.415	1.707	1.488	2.072	1.395	1.266	1.651	1.485
SAMD9	1.718	1.476	1.563	1.972	2.005	1.699	1.744	1.802	1.673
SPATS2L	1.888	1.550	2.066	2.435	2.223	2.002	2.157	1.880	1.884
TNFSF10	0.978	1.508	1.918	1.596	2.152	1.822	1.176	2.043	1.862
USP18	3.564	3.171	2.863	4.084	4.094	3.009	3.755	3.742	2.857
XAF1	1.802	1.600	1.723	2.105	1.843	1.923	1.877	1.822	1.863

Supplementary Table S3: List of canonical pathways of common differentially expressed genes (DEGs) from EV-A71-infected PBMCs at 6-24 hpi

No.	Canonical Pathways	-log(*p*-value)	Ratio	Molecules
1	Interferon Signaling	10.40	1.67E-01	*IFITM3,MX1,IFI35,IFI6,IFITM1,ISG15*
2	Activation of IRF by Cytosolic Pattern Recognition Receptors	7.09	8.20E-02	*DHX58,IFIH1,IRF7,DDX58,ISG15*
3	Role of RIG-I-like Receptors in Antiviral Innate Immunity	6.01	9.52E-02	*DHX58,IFIH1,IRF7,DDX58*
4	Role of Pattern Recognition Receptors in Recognition of Bacteria and Viruses	4.04	3.05E-02	*IFIH1,IRF7,DDX58,EIF2AK2*
5	Salvage Pathways of Pyrimidine Ribonucleotides	1.89	2.11E-02	*EIF2AK2,APOBEC3A*
6	Salvage Pathways of Pyrimidine Deoxyribonucleotides	1.84	1.25E-01	*APOBEC3A*
7	Guanosine Nucleotides Degradation III	1.67	8.33E-02	*NT5C3A*
8	Urate Biosynthesis/Inosine 5'-phosphate Degradation	1.63	7.69E-02	*NT5C3A*
9	Adenosine Nucleotides Degradation II	1.57	6.67E-02	*NT5C3A*
10	Purine Nucleotides Degradation II (Aerobic)	1.49	5.56E-02	*NT5C3A*
11	Role of Lipids/Lipid Rafts in the Pathogenesis of Influenza	1.45	5.00E-02	*RSAD2*
12	NAD Salvage Pathway II	1.43	4.76E-02	*NT5C3A*

Supplementary Table S4: List of significant differentially expressed genes (DEGs) in EV-A71-infected PBMCs at 24 hpi

Gene	Log_2-(fold change of infected over heat-inactivated)		
	Mild EV-A71	Moderate EV-A71	Severe EV-A71
AGRN	1.194	1.330	1.160
APOBEC3A	3.195	3.890	3.410
APOE	-2.167	-3.071	-4.181
ATF3	1.251	1.413	1.276
AXL	1.385	1.244	1.285
B3GNT2	1.134	1.274	1.060
CCL3	1.319	1.582	1.640
CCL7	2.739	2.770	2.871
CCL8	3.567	4.017	3.842
CCR2	-1.588	-2.348	-1.684
CFAP43	4.377	4.627	4.269
CLEC10A	-1.242	-1.862	-1.137
CTSL	1.696	2.066	1.719
CXCL10	3.666	3.480	3.405
CYP19A1	5.404	6.382	5.805
DDX58	1.860	1.934	1.855
DDX60L	1.786	1.775	1.754
DHX58	1.645	1.661	1.657
DUSP5	0.998	1.218	1.309
EIF2AK2	1.751	1.927	1.926
EPSTI1	1.373	1.407	1.325
ERICH3	4.493	4.872	4.839
GMPR	2.602	2.309	2.224
HELZ2	2.036	2.311	2.300
HERC5	2.361	2.006	2.033
HERC6	2.358	2.512	2.534
HESX1	4.170	4.071	3.718
HPSE	1.750	1.859	1.633
IFI27	2.644	3.161	2.957
IFI35	1.530	1.479	1.351
IFI44	2.315	2.293	2.234
IFI44L	2.582	2.653	2.640
IFI6	2.314	2.355	2.094
IFIH1	1.560	1.619	1.546
IFIT5	1.445	1.487	1.512
IFITM1	2.207	2.317	2.317
IFITM3	1.849	1.876	1.755

IGFBP4	1.421	1.164	1.223
IL1RN	2.456	2.534	2.299
IL27	2.644	2.521	2.321
IRF7	1.701	1.741	1.742
ISG15	3.472	3.134	3.058
ISG20	2.368	2.186	2.140
KITLG	1.836	2.548	1.961
LAG3	1.740	1.569	1.849
LAMP3	1.114	1.260	1.506
LGALS3BP	1.127	1.213	1.304
LILRA5	1.510	1.794	1.746
LY6E	1.801	1.884	1.747
MX1	2.265	2.326	2.242
MX2	2.145	2.321	2.248
NEURL3	2.961	3.867	3.692
NEXN	2.611	2.154	2.004
NT5C3A	2.173	2.012	2.002
OASL	2.331	2.529	2.433
OTOF	2.797	3.475	3.244
PADI2	-1.964	-2.652	-2.116
PHF11	1.047	2.025	2.233
PLSCR1	1.828	1.849	1.746
PNPT1	1.718	1.578	1.600
RIN2	1.145	1.553	1.231
RSAD2	3.670	3.778	3.653
RTP4	1.707	1.395	1.485
SAMD4A	1.198	1.340	1.254
SAMD9	1.563	1.699	1.673
SAMD9L	1.539	1.631	1.526
SLC38A5	1.587	1.798	1.754
SPATS2L	2.066	2.002	1.884
TNFSF10	1.918	1.822	1.862
USP18	2.863	3.009	2.857
XAF1	1.723	1.923	1.863
ZBP1	1.459	1.541	1.535
CHI3L1	-1.672	-2.382	N.A.
EPHB2	0.984	1.012	N.A.
HSPA1A	1.287	1.001	N.A.
IL1R2	1.574	1.109	N.A.
RASGEF1B	1.127	1.194	N.A.
ABTB2	N.A.	1.930	1.891
ALDH1A1	N.A.	-1.559	-1.741
ALDH2	N.A.	-1.291	-1.430

ANKRD1	N.A.	3.335	3.245
ARNT2	N.A.	2.335	2.118
C19orf66	N.A.	1.094	1.145
CCL4	N.A.	1.241	1.539
CD300E	N.A.	2.072	1.738
CD9	N.A.	-1.004	-1.546
CLMP	N.A.	3.837	3.592
FBP1	N.A.	-1.194	-1.283
FSCN1	N.A.	1.174	1.103
IRG1	N.A.	2.175	1.995
ITGB8	N.A.	1.932	2.388
LTA4H	N.A.	-1.325	-1.423
MDK	N.A.	2.054	2.072
NFAM1	N.A.	-1.043	-1.037
PLBD1	N.A.	-1.145	-1.165
SERPINA1	N.A.	-1.193	-1.228
SERPINB2	N.A.	1.169	1.735
SSTR3	N.A.	1.631	1.831
TNFAIP6	N.A.	1.611	1.699
TREM2	N.A.	-1.444	-2.012
WASH5P	N.A.	5.643	5.554
OLR1	-1.552	N.A.	-1.763
DYNLT1	1.195	N.A.	N.A.
GCH1	1.016	N.A.	N.A.
HSPA1B	1.109	N.A.	N.A.
MARCKS	1.186	N.A.	N.A.
MT2A	1.392	N.A.	N.A.
NMI	0.964	N.A.	N.A.
NUPR1	2.771	N.A.	N.A.
PGAP1	2.100	N.A.	N.A.
PPBP	1.347	N.A.	N.A.
RNASE1	1.655	N.A.	N.A.
RTCB	1.038	N.A.	N.A.
SCIN	1.662	N.A.	N.A.
SPP1	1.253	N.A.	N.A.
ATP13A2	N.A.	1.481	N.A.
CCL24	N.A.	-1.378	N.A.
CD1D	N.A.	-1.425	N.A.
CXCL13	N.A.	2.369	N.A.
FCN1	N.A.	-1.242	N.A.
IDO1	N.A.	1.318	N.A.
MSR1	N.A.	1.128	N.A.
MYCL	N.A.	-1.278	N.A.

MYO10	N.A.	1.705	N.A.
PML	N.A.	1.156	N.A.
RNF19B	N.A.	1.097	N.A.
SCARB1	N.A.	-1.575	N.A.
SEMA4A	N.A.	0.997	N.A.
SLC1A4	N.A.	1.013	N.A.
SLC39A8	N.A.	1.525	N.A.
SLC7A8	N.A.	1.431	N.A.
SPARC	N.A.	-2.077	N.A.
SRGAP2	N.A.	1.143	N.A.
TIFAB	N.A.	-1.290	N.A.
XCR1	N.A.	1.485	N.A.
ABCC3	N.A.	N.A.	-1.363
APOL4	N.A.	N.A.	-1.196
CCL4L1	N.A.	N.A.	1.636
CEBPA	N.A.	N.A.	-1.142
CKB	N.A.	N.A.	2.148
CXCL1	N.A.	N.A.	1.304
CXCL3	N.A.	N.A.	1.036
CXCL5	N.A.	N.A.	1.526
FN1	N.A.	N.A.	-1.274
IER5L	N.A.	N.A.	-1.502
IL-1β	N.A.	N.A.	1.083
IL-6	N.A.	N.A.	3.212
PHF11	N.A.	N.A.	1.012
PRAM1	N.A.	N.A.	-1.526
PYCR1	N.A.	N.A.	2.920
RGS1	N.A.	N.A.	1.075

Supplementary Table S5: List of canonical pathways from differentially expressed genes (DEGs) of PBMC-infected EV-A71 isolates at 24 hpi

No.	Canonical Pathways	Activation *z*-score			-log(*p*-value)			Molecules
		Mild EV-A71	Moderate EV-A71	Severe EV-A71	Mild EV-A71	Moderate EV-A71	Severe EV-A71	
1	Interferon Signalling	2.449	2.449	2.449	7.894	7.143	7.322	*IFITM3, MX1, IFI6, IFI35, IFITM1, ISG15*
2	LXR/RXR Activation	-2.000	-2.236	-2.449	2.651	4.029	4.196	*APOE, IL1RN, IL-1b, SERPINA1, IL-6, CCL7*
3	Role of Pattern Recognition Receptors in Recognition of Bacteria and Viruses	2.000	2.000	2.449	2.526	2.084	4.004	*IFIH1, IRF7, DDX58, IL-1b, IL-6, EIF2AK2*
4	IL-6 Signalling	N.A.	2.000	2.236	1.638	2.050	3.010	*TNFAIP6, IL1RN, CYP19A1, IL1-b, IL-6*
5	Role of RIG-I-like Receptors in Antiviral Innate Immunity	1.000	1.000	1.000	4.413	3.925	4.042	*IFIH1, DHX58, IRF7, DDX58*
6	Activation of IRF by Cytosolic Pattern Recognition Receptors	0.816	0.816	1.134	6.479	5.741	7.293	*IFIH1, DHX58, IRF7, ZBP1, DDX58, IL-6, ISG15*
7	Neuroinflammation Signaling Pathway	N.A.	1.000	1.633	0.806	0.960	2.142	*CXCL10, IRF7, TREM2, IL-1b, IL-6, CCL3*
8	Role of IL-17F in Allergic Inflammatory Airway Diseases	N.A.	N.A.	2.449	1.809	2.682	8.464	*CXCL10, CCL4, IL-1b, CXCL1, CXCL5, IL-6, CCL7*
9	TREM-1 Signalling	N.A.	N.A.	2.236	1.396	1.174	4.327	*CXCL3, IL-1b, IL6, CCL3, CCL7*
10	Dendritic Cell Maturation	N.A.	0.000	1.342	0.244	1.586	2.399	*IL1RN, FSCN1, TREM2, IL-1b, IL-6*

Supplementary Table S6: Information of primers used for gene expression

Gene	Direction	Sequence (5' to 3')
CXCL3	Forward	CGCCCAAACCGAAGTCATAG
	Reverse	GCTCCCCTTGTTCAGTATCTTTT
IL-1β	Forward	AAATACCTGTGGCCTTGGGC
	Reverse	TTTGGGATCTACACTCTCCAGCT
IL-6	Forward	CACAGACAGCCACTCACCTCTTCAGAACGA
	Reverse	ACCAGTGATTTTCACCAGGCAAGTCTC
CCL2	Forward	CAGCCAGATGCAATCAATGCC
	Reverse	TGGAATCCTGAACCCACTTCT
CCL7	Forward	TGCTCAGCCAGTTGGGATTA
	Reverse	GGACAGTGGCTACTGGTGGT
CCL3	Forward	TCAGACTTCAGAAGGACACGG
	Reverse	CTGCATGATTCTGAGCAGGTG
TLR7	Forward	TCCTTGGGGCTAGATGGTTTC
	Reverse	TCCACGATCACATGGTTCTTTG
TNFα	Forward	TGCTTGTTCCTCAGCCTCTT
	Reverse	GGAAGACCCCTCCCAGATAG
GAPDH	Forward	CCACATCGCTCAGACACCAT
	Reverse	GGCAACAATATCCACTTTACCAGAGT
